# Spherical Fuzzy Logarithmic Aggregation Operators Based on Entropy and Their Application in Decision Support Systems

**DOI:** 10.3390/e21070628

**Published:** 2019-06-26

**Authors:** Yun Jin, Shahzaib Ashraf, Saleem Abdullah

**Affiliations:** 1 School of Economic and Management, Nanjing University of Aeronautics and Astronautics, Nanjing 211106, China; 2 Department of Mathematics, Abdul Wali Khan University, Mardan 23200, Pakistan

**Keywords:** spherical fuzzy sets, logarithmic spherical operational laws, logarithmic spherical aggregation operators, entropy, multi-criteria group decision making (MCGDM) problems

## Abstract

Keeping in view the importance of new defined and well growing spherical fuzzy sets, in this study, we proposed a novel method to handle the spherical fuzzy multi-criteria group decision-making (MCGDM) problems. Firstly, we presented some novel logarithmic operations of spherical fuzzy sets (SFSs). Then, we proposed series of novel logarithmic operators, namely spherical fuzzy weighted average operators and spherical fuzzy weighted geometric operators. We proposed the spherical fuzzy entropy to find the unknown weights information of the criteria. We study some of its desirable properties such as idempotency, boundary and monotonicity in detail. Finally, the detailed steps for the spherical fuzzy decision-making problems were developed, and a practical case was given to check the created approach and to illustrate its validity and superiority. Besides this, a systematic comparison analysis with other existent methods is conducted to reveal the advantages of our proposed method. Results indicate that the proposed method is suitable and effective for the decision process to evaluate their best alternative.

## 1. Introduction

The complication of a system is growing every day in real life and getting the finest option from the set of possible ones is difficult for the decision makers. To attain a single objective is difficult to summarize but not incredible. Many organizations found difficulties with setting motivations, goals and opinions’ complications. Thus, organizational decisions simultaneously include numerous objectives, whether they regard individuals or committees. This reflection suggests, according to criteria solved optionally, restricting each decision maker to attain an ideal solution-optimum under each criterion involved in practical problems. Consequently, the decision maker is more concentrated to establish more applicable and reliable techniques to find the best options.

To handle the ambiguity and uncertainty data in decision-making problems, the classical or crisp methods cannot be always effective. Thus, dealing with such uncertain situations, Zadeh [1] in 1965 presented the idea of the fuzzy set. Zadeh assigns membership grades to elements of a set in the interval [0,1] by offering the idea of fuzzy sets (FSs). Zadeh’s work in this direction is remarkable as many of the set theoretic properties of crisp cases were defined for fuzzy sets. Fuzzy set theory got the attention of researchers and found its applications in decision science [2], artificial intelligence [3], and medical diagnosis [4], and its enormous applications are discussed in [5].

After many applications of fuzzy set theory, Atanassov observed that there are many shortcomings in this theory and introduced the notion of intuitionistic fuzzy sets [6] to generalize the concept of Zadeh’s fuzzy set. In intuitionistic fuzzy sets, each element is expressed by an ordered pair, and each pair is characterized by membership and non-membership grades on the condition that the sum of their grades are less than or equal to 1. During the last few decades, the intuitionistic fuzzy sets (IFSs) are fruitful and broadly utilized by researchers to grasp the ambiguity and imprecision data. To cumulate all the executive of criteria for alternatives, aggregation operators play a vital role throughout the information merging procedure. Xu [7] presented a weighted averaging operator while Xu and Yager [8] developed a geometric aggregation operator for aggregating the different intuitionistic fuzzy numbers. Verma [9] in 2015 proposed the generalized Bonferroni mean operator and, in [10], Verma and Sharma proposed the measure of inaccuracy using intuitionistic fuzzy information. Deschrijver [11] developed the IFS representation of t-norms and t-conorms. In some decision theories, the decision makers deal with the situation of particular attributes where values of their summation of membership degrees exceeds 1. In such conditions, IFS has no ability to obtain any satisfactory result. To overcome this situation, Yager [12] developed the idea of a Pythagorean fuzzy set (PyFS) as a generalization of IFS, which satisfies the fact that the value of square summation of its membership degrees is less then or equal to 1. Clearly, PyFS is more flexible than IFS to deal with the imprecision and ambiguity in the practical multi-criteria decision-making (MCDM) problems. Zhang and Xu [13] established an extension of TOPSIS to MCDM with PyFS information. The error for the proof of distance measure in Zhang and Xu [13] has been pointed out by Yang et al. [14]. For MCDM problems in a Pythagorean fuzzy environment, Yager and Abbasov [15] developed a series of aggregation operators. Peng and Yang [16] explained their relationship among these aggregation operators and established the superiority and inferiority ranking (SIR) for the multi-criteria group decision-making (MCGDM) method. Using Einstein operation, Garg [17] generalized Pythagorean fuzzy information aggregation. Gou et al. [18] studied many Pythagorean fuzzy functions and investigated their fundamental properties such as continuity, derivatively, and differentiability in detail. Zhang [19] put forward a ranked qualitative flexible (QUALIFLEX) multi-criteria approach with the closeness index-based ranking methods for multi-criteria Pythagorean fuzzy decision analysis. Zeng et al. [20] explored a hybrid method for Pythagorean fuzzy MCDM. Zeng [21], applied the Pythagorean fuzzy probabilistic OWA (PFPOWA) operator for MAGDM problems. For more study, we refer to [22,23,24].

IFS theory and PyFS theory have been successfully applied in different areas, but there are situations that cannot be represented by it in real life, such as voting, we may face human opinions involving more answers of the type: yes, abstain, no and refusal (for example, in a democratic election station, the council issues’ 500 voting papers for a candidate. The voting results are divided into four groups accompanied with the number of papers that are vote for (251), abstain (99), vote against (120) and refusal of voting (30). Here, group abstain means that the voting paper is a white paper rejecting both agree and disagree for the candidate but still takes the vote, group refusal of voting is either invalid voting papers or did not take the vote. The candidate is successful because the number of support papers is over 50% (i.e., 250).

However, at least five people said later on in their blogs that they support the candidate in the last moment because they find that the support number seems larger than the against number. Such kind of examples (in which the number of abstains is a key factor and the group refusal of voting indeed exists) happened in reality and IFS and PyFS could not handle it). Thus, Cuong [25] proposed a new notion named picture fuzzy sets, which is an extension of fuzzy sets and intuitionistic fuzzy sets. Picture fuzzy sets give three membership degrees of an element named the positive membership degree, the neutral membership degree, and the negative membership degree, respectively. The picture fuzzy set solved the voting problem successfully, and is applied to clustering [26], fuzzy inference [27], and decision-making [28,29,30,31,32,33,34,35].

The neutrosophic set is another important generalizations of the classic set, fuzzy set, intuitionistic fuzzy set and picture fuzzy set to deal with uncertainties in decision-making problems. Many authors contributed in the decision-making theory using neutrosophic information. Ashraf [36] proposed the logarithmic hybrid aggregation operators for single value neutrosophic sets. Dragan et al. [37] proposed the novel approach for the selection of power generation technology using the combinative distance-based assessment (CODAS) method. Many decision-making approaches like [38,39] make important contributions using neutrosophic information.

The picture fuzzy set becomes more famous by introducing various kinds of aggregation operators. However, it has a shortcoming in that it is only valid for the environment whose sum of degrees is less than or equal to one. However, in day-to-day life, there are many situations where this condition is ruled out. For instance, if a person giving their preference in the form of positive, neutral and negative membership degrees towards a particular object is 0.7, 0.3 and 0.5, then clearly this situation is not handling with picture fuzzy set. In order to resolve it, Ashraf [40] proposed the notion of the spherical fuzzy set. For instance, corresponding to the above-considered example, we see that ${\left(0.7\right)}^{2}+{\left(0.3\right)}^{2}+{\left(0.5\right)}^{2}=0.94$ and hence a spherical fuzzy set (SFS) is an extension of the existing extensions of fuzzy set theories. In the spherical fuzzy set, each element can be written in the form of triplet component, and each pair is categorized by a positive membership degree, a neutral membership degree and a negative membership degree such that the sum of their square is less than or equal to one. Ashraf [41] proposed some series of spherical aggregation operators using t-norm and t-conorm and gave its applications to show the effectiveness of proposed operators. Ashraf in [42] proposed the GRA method based on a spherical linguistic fuzzy Choquet integral environment and gave its application. For more study, we refer to [43,44,45].

The logarithmic operations being good alternatives, compared with the algebraic operations, have the potential to offer similar smooth estimations as the algebraic operations. However, there is little investigation on logarithmic operations on the IFSs and PyFSs. Motivated by these ideas, we develop a spherical fuzzy MCDM method based on the logarithmic aggregation operators, with the logarithmic operations of the spherical fuzzy sets (SFSs) handling spherical fuzzy MCDM within SFSs.

Thus, the goal of this article is to propose the decision-making method for MCDM problems in which there exist the interrelationships among the criteria. The contributions of this study are:

- We develop some novel logarithmic operations for spherical fuzzy sets, which can overcome the weaknesses of algebraic operations and capture the relationship between various SFSs.
- We extend logarithmic operators to logarithmic spherical fuzzy operators, namely logarithmic spherical fuzzy weighted averaging (L-SFWA), logarithmic spherical fuzzy ordered weighted averaging (L-SFOWA), logarithmic spherical fuzzy hybrid weighted averaging (L-SFHWA), logarithmic spherical fuzzy weighted geometric (L-SFWG), logarithmic spherical fuzzy ordered weighted geometric (L-SFOWG) and logarithmic spherical fuzzy hybrid weighted geometric (L-SFHWG) to SFSs, which can overcome the algebraic operators’ drawbacks.
- We develop the spherical fuzzy entropy for spherical fuzzy information, which can help to find the unknown weights information of the criteria.
- We develop an algorithm to deal with multi-attribute decision-making problems using spherical fuzzy information.
- To show the effectiveness and reliability of the proposed spherical fuzzy logarithmic aggregation operators, the application of the proposed operator in emerging technology enterprises is developed.
- Results indicate that the proposed technique is more effective and gives more accurate output as compared to existing studies.

In order to attain the research goal that has been stated above, the organization of this article is offered as: Section 2 concentrates on some basic notions and operations of existing extensions of fuzzy set theories and also some discussion to propose the spherical fuzzy entropy. Section 3 presents some novel logarithmic operational laws of SFSs. Section 4 defines the logarithmic aggregation operators for SFNs and discusses its properties. Section 5 presents an approach for handling the spherical fuzzy MCDM problem based on the proposed logarithmic operators. Section 5.1 uses an application case to verify the novel method and Section 5.2 presents the comparison study about algebraic and logarithmic aggregation operators. Section 6 concludes the study.

## 2. Preliminaries

The concepts and basic operations of existing extensions of fuzzy sets are recalled in this section, and they are the foundation of this study.

**Definition** **1** ([27])**.** *A mapping $\hat{T}:\Theta \times \Theta \to \Theta$ is said to be triangular-norm if each element $\;\hat{T}$ satisfies that:*

*(1) $\hat{T}$ is commutative, monotonic and associative,*

(2) $\hat{T}\left(v^{*},1\right)=v^{*},$ each $v^{*}\in \hat{T},$

*where Θ=[0,1] is the unite interval.*

**Definition** **2** ([27])**.** *A mapping $\hat{S}:\Theta \times \Theta \to \Theta$ is said to be triangular-conorm if each element $\;\hat{S}$ satisfies that*

*(1) $\hat{S}$ is commutative, monotonic and associative,*

(2) $\hat{S}\left(v^{*},0\right)=v^{*},$ each $v^{*}\in \hat{S},$

*where Θ=[0,1] is the unite interval.*

**Figure 1:**
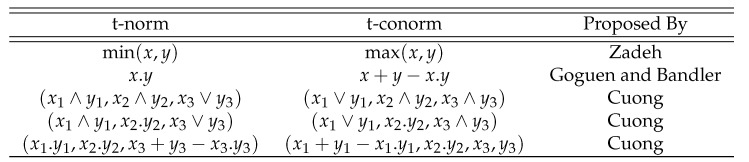
Basic Norms Operations.

As different norms are the curial elements for proposing aggregation operators in fuzzy set theory, here we enlist some basic norms operations for fuzzy sets in Figure 1.

**Figure 2:**
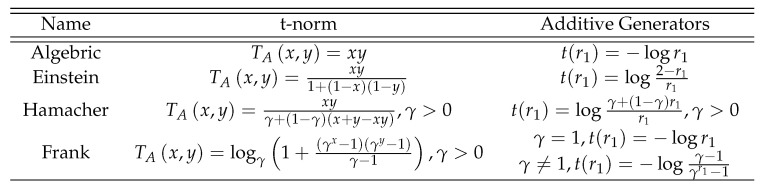
T-norm with its Generators.

**Figure 3:**
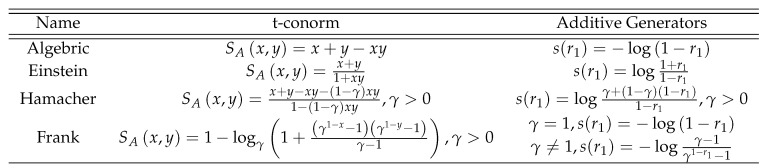
T-conorm with its Generators.

Now, we enlist different types of norms with its generators too in Figure 2 and Figure 3.

**Definition** **3** ([15])**.** *For a set ℜ, by a Pythagorean fuzzy set in ℜ, we mean a structure*

$$\varepsilon =\left\{\langle P_{\sigma }\left({\overset{ˇ}{r}}_{\gamma }\right),N_{\sigma }\left({\overset{ˇ}{r}}_{\gamma }\right)\rangle |{\overset{ˇ}{r}}_{\gamma }\in ℜ\right\},$$

*in which $P_{\sigma }:ℜ\to \Theta$ and $N_{\sigma }:ℜ\to \Theta$ indicate that the positive and negative grades in ℜ,$\Theta =\left[0,1\right]$ are the unit intervals. In addition, the following condition satisfied by $\rho_{\sigma }$ and $N_{\sigma }$ is $0\leq P_{\sigma }^{2}\left({\overset{ˇ}{r}}_{\gamma }\right)+N_{\sigma }^{2}\left({\overset{ˇ}{r}}_{\gamma }\right)\leq 1$; for all ${\overset{ˇ}{r}}_{⋎}\in ℜ.$ Then, ε is said to be a Pythagorean fuzzy set in ℜ.*

**Definition** **4** ([25])**.** *For a set ℜ, by a picture fuzzy set in ℜ, we mean a structure*

$$\varepsilon =\left\{\langle P_{\sigma }\left({\overset{ˇ}{r}}_{\gamma }\right),I_{\sigma }\left({\overset{ˇ}{r}}_{\gamma }\right),N_{\sigma }\left({\overset{ˇ}{r}}_{\gamma }\right)\rangle |{\overset{ˇ}{r}}_{\gamma }\in ℜ\right\},$$

*in which $P_{\sigma }:ℜ\to \Theta ,$$I_{\sigma }:ℜ\to \Theta$ and $N_{\sigma }:ℜ\to \Theta$ indicate that the positive, neutral and negative grades in ℜ, $\Theta =\left[0,1\right]$ are the unit intervals. In addition, the following condition satisfied by $P_{\sigma },I_{\sigma }$ and $N_{\sigma }$ is $0\leq P_{\sigma }\left({\overset{ˇ}{r}}_{\gamma }\right)+I_{\sigma }\left({\overset{ˇ}{r}}_{\gamma }\right)+N_{\sigma }\left({\overset{ˇ}{r}}_{\gamma }\right)\leq 1$, for all ${\overset{ˇ}{r}}_{\gamma }\in ℜ.$ Then, ε is said to be a picture fuzzy set in ℜ.*

**Definition** **5** ([40])**.** *For a set ℜ, by a spherical fuzzy set in ℜ, we mean a structure*

$$\varepsilon =\left\{\langle P_{\sigma }\left({\overset{ˇ}{r}}_{\gamma }\right),I_{\sigma }\left({\overset{ˇ}{r}}_{\gamma }\right),N_{\sigma }\left({\overset{ˇ}{r}}_{\gamma }\right)\rangle |{\overset{ˇ}{r}}_{\gamma }\in ℜ\right\},$$

*in which $P_{\sigma }:ℜ\to \Theta ,$$I_{\sigma }:ℜ\to \Theta$ and $N_{\sigma }:ℜ\to \Theta$ indicate that the positive, neutral and negative grades in ℜ, $\Theta =\left[0,1\right]$ are the unit intervals. In addition, the following condition satisfies by $P_{\sigma },I_{\sigma }$ and $N_{\sigma }$ is $0\leq P_{\sigma }^{2}\left({\overset{ˇ}{r}}_{\gamma }\right)+I_{\sigma }^{2}\left({\overset{ˇ}{r}}_{\gamma }\right)+N_{\sigma }^{2}\left({\overset{ˇ}{r}}_{\gamma }\right)\leq 1$, for all ${\overset{ˇ}{r}}_{\gamma }\in ℜ.$ Then, ε is said to be a spherical fuzzy set in ℜ.*

*$\chi_{\sigma }\left({\overset{ˇ}{r}}_{\gamma }\right)=\sqrt{1-\left(P_{\sigma }^{2}\left({\overset{ˇ}{r}}_{\gamma }\right)+I_{\sigma }^{2}\left({\overset{ˇ}{r}}_{\gamma }\right)+N_{\sigma }^{2}\left({\overset{ˇ}{r}}_{\gamma }\right)\right)}$ is said to be a refusal degree of ${\overset{ˇ}{r}}_{\gamma }$ in ℜ, for SFS $\left\{\langle P_{\sigma }\left({\overset{ˇ}{r}}_{\gamma }\right),I_{\sigma }\left({\overset{ˇ}{r}}_{\gamma }\right),N_{\sigma }\left({\overset{ˇ}{r}}_{\gamma }\right)\rangle |{\overset{ˇ}{r}}_{\gamma }\in ℜ\right\}$, which is triple components $\langle P_{\sigma }\left({\overset{ˇ}{r}}_{\gamma }\right),I_{\sigma }\left({\overset{ˇ}{r}}_{\gamma }\right),andN_{\sigma }\left({\overset{ˇ}{r}}_{\gamma }\right)\rangle$ is said to SFN denoted by $e=\langle P_{e},I_{e},N_{e}\rangle$, where $P_{e},I_{e}$ and $N_{e}\in \left[0,1\right]$, with the condition that: $0\leq P_{e}^{2}+I_{e}^{2}+N_{e}^{2}\leq 1.$*

Ashraf and Abdullah [40] proposed the basic operations of spherical fuzzy set as follows:

**Definition** **6.** *For any two SFNs, $\varepsilon_{\rho }=\langle P_{\xi_{\rho }}\left({\overset{ˇ}{r}}_{\gamma }\right),I_{\xi_{\rho }}\left({\overset{ˇ}{r}}_{\gamma }\right),N_{\xi_{\rho }}\left({\overset{ˇ}{r}}_{\gamma }\right)\rangle$ and $\varepsilon_{q}=\langle P_{\xi_{q}}\left({\overset{ˇ}{r}}_{\gamma }\right),I_{\xi_{q}}\left({\overset{ˇ}{r}}_{\gamma }\right),N_{\xi_{q}}\left({\overset{ˇ}{r}}_{\gamma }\right)\rangle$ in ℜ. The union, intersection and compliment are proposed as:*

*(1) $\varepsilon_{\rho }\subseteq \varepsilon_{q}$iff$\forall {\overset{ˇ}{r}}_{\gamma }\in ℜ,$$P_{\xi_{\rho }}\left({\overset{ˇ}{r}}_{\gamma }\right)\leq P_{\xi_{q}}\left({\overset{ˇ}{r}}_{\gamma }\right),$$I_{\xi_{\rho }}\left({\overset{ˇ}{r}}_{\gamma }\right)\leq I_{\xi_{q}}\left({\overset{ˇ}{r}}_{\gamma }\right)$ and $N_{\xi_{\rho }}\left({\overset{ˇ}{r}}_{\gamma }\right)\geq N_{\xi_{q}}\left({\overset{ˇ}{r}}_{\gamma }\right)$;*

*(2) $\varepsilon_{\rho }=\varepsilon_{q}$iff$\varepsilon_{\rho }\subseteq \varepsilon_{q}$ and $\varepsilon_{q}\subseteq \varepsilon_{\rho }$;*

*(3) $\varepsilon_{\rho }\cup \varepsilon_{q}=\langle \max\left(P_{\xi_{\rho }},P_{\xi_{q}}\right),\;\min\left(I_{\xi_{\rho }},I_{\xi_{q}}\right),\;\min\left(N_{\xi_{\rho }},N_{\xi_{q}}\right)\rangle ;$*

*(4) $\varepsilon_{\rho }\cap \varepsilon_{q}=\langle \min\left(P_{\xi_{\rho }},P_{\xi_{q}}\right),\;\min\left(I_{\xi_{\rho }},I_{\xi_{q}}\right),\;\max\left(N_{\xi_{\rho }},N_{\xi_{q}}\right)\rangle ;$*

*(5) $\varepsilon_{\rho }=\langle N_{\xi_{\rho }},I_{\xi_{\rho }},P_{\xi_{\rho }}\rangle .$*

**Definition** **7.** *For any two SFNs, $\varepsilon_{\rho }=\langle P_{\xi_{\rho }}\left({\overset{ˇ}{r}}_{\gamma }\right),I_{\xi_{\rho }}\left({\overset{ˇ}{r}}_{\gamma }\right),N_{\xi_{\rho }}\left({\overset{ˇ}{r}}_{\gamma }\right)\rangle$ and $\varepsilon_{q}=\langle P_{\xi_{q}}\left({\overset{ˇ}{r}}_{\gamma }\right),I_{\xi_{q}}\left({\overset{ˇ}{r}}_{\gamma }\right),N_{\xi_{q}}\left({\overset{ˇ}{r}}_{\gamma }\right)\rangle$ in ℜ and β≥0; then, the operations of SFNs are proposed as*

*(1) $\varepsilon_{\rho }⊕\varepsilon_{q}=\left\{\sqrt{P_{\xi_{\rho }}^{2}+P_{\xi_{q}}^{2}-P_{\xi_{\rho }}^{2}\cdot P_{\xi_{q}}^{2}},\;I_{\xi_{\rho }}\cdot I_{\xi_{q}},\;N_{\xi_{\rho }}\cdot N_{\xi_{q}}\right\};$*

*(2) $\beta \cdot \varepsilon_{\rho }=\left\{\sqrt{1-{\left(1-P_{\xi_{\rho }}^{2}\right)}^{\beta }},\;{\left(I_{\xi_{\rho }}\right)}^{\beta },\;{\left(N_{\xi_{\rho }}\right)}^{\beta }\right\};$*

*(3) $\varepsilon_{\rho }⊗\varepsilon_{q}=\left\{P_{\xi_{\rho }}\cdot P_{\xi_{q}},\;I_{\xi_{\rho }}\cdot I_{\xi_{q}},\sqrt{N_{\xi_{\rho }}^{2}+N_{\xi_{q}}^{2}-N_{\xi_{\rho }}^{2}\cdot N_{\xi_{q}}^{2}}\right\};$*

*(4) $\varepsilon_{\rho }^{\beta }=\left\{{\left(P_{\xi_{\rho }}\right)}^{\beta },\;{\left(I_{\xi_{\rho }}\right)}^{\beta },\sqrt{1-{\left(1-N_{\xi_{\rho }}^{2}\right)}^{\beta }}\right\}.$*

*(5) $\beta^{\varepsilon_{\rho }}=\left\{\begin{array}{cc} \left(\beta^{\sqrt{1-\rho_{\xi_{\rho }}^{2}}},\sqrt{1-\beta^{2I_{\xi_{\rho }}}},\sqrt{1-\beta^{2N_{\xi_{\rho }}}}\right) & if\;\beta \in \left(0,1\right),\\ \left({\left(\frac{1}{\beta }\right)}^{\sqrt{1-\rho_{\xi_{\rho }}^{2}}},\sqrt{1-{\left(\frac{1}{\beta }\right)}^{2I_{\xi_{\rho }}}},\sqrt{1-{\left(\frac{1}{\beta }\right)}^{2N_{\xi_{\rho }}}}\right) & if\;\beta \geq 1. \end{array}\right.$*

Ashraf and Abdullah [40] introduced some properties based on Definition 7 as follows:

**Definition** **8.** *For any three SFNs, $\varepsilon_{\rho }=\langle P_{\xi_{\rho }}\left({\overset{ˇ}{r}}_{\gamma }\right),I_{\xi_{\rho }}\left({\overset{ˇ}{r}}_{\gamma }\right),N_{\xi_{\rho }}\left({\overset{ˇ}{r}}_{\gamma }\right)\rangle$, $\varepsilon_{q}=\langle P_{\xi_{q}}\left({\overset{ˇ}{r}}_{\gamma }\right),I_{\xi_{q}}\left({\overset{ˇ}{r}}_{\gamma }\right),N_{\xi_{q}}\left({\overset{ˇ}{r}}_{\gamma }\right)\rangle$ and $\varepsilon_{l}=\langle P_{\sigma_{l}}\left({\overset{ˇ}{r}}_{\gamma }\right),I_{\sigma_{l}}\left({\overset{ˇ}{r}}_{\gamma }\right),N_{\sigma_{l}}\left({\overset{ˇ}{r}}_{\gamma }\right)\rangle$ in ℜ and $\beta_{1},\beta_{2}\geq 0.$ Then,*

*(1) $\varepsilon_{\rho }⊕\varepsilon_{q}=\varepsilon_{q}⊕\varepsilon_{\rho };$*

*(2) $\varepsilon_{\rho }⊗\varepsilon_{q}=\varepsilon_{q}⊗\varepsilon_{\rho };$*

*(3) $\beta_{1}\left(\varepsilon_{\rho }⊕\varepsilon_{q}\right)=\beta_{1}\varepsilon_{\rho }⊕\beta_{1}\varepsilon_{q},$$\;\beta_{1}>0;$*

*(4) ${\left(\varepsilon_{\rho }⊗\varepsilon_{q}\right)}^{\beta_{1}}=\varepsilon_{\rho }⊗\varepsilon_{q},$$\;\beta_{1}>0;$*

*(5) $\beta_{1}\varepsilon_{\rho }⊕\beta_{2}\varepsilon_{\rho }=\left(\beta_{1}+\beta_{2}\right)\varepsilon_{\rho },$$\beta_{1}>0,$$\beta_{2}>0;$*

*(6) $\varepsilon_{\rho }⊗\varepsilon_{\rho }=\varepsilon_{\rho },$$\beta_{1}>0,$$\beta_{2}>0;$*

*(7) $\left(\varepsilon_{\rho }⊕\varepsilon_{q}\right)⊕\varepsilon_{l}=\varepsilon_{\rho }⊕\left(\varepsilon_{q}⊕\varepsilon_{l}\right);$*

*(8) $\left(\varepsilon_{\rho }⊗\varepsilon_{q}\right)⊗\varepsilon_{l}=\varepsilon_{\rho }⊗\left(\varepsilon_{q}⊗\varepsilon_{l}\right).$*

**Definition** **9.** *For any SFN, $\varepsilon_{\rho }=\langle P_{\xi_{\rho }}\left({\overset{ˇ}{r}}_{\gamma }\right),I_{\xi_{\rho }}\left({\overset{ˇ}{r}}_{\gamma }\right),N_{\xi_{\rho }}\left({\overset{ˇ}{r}}_{\gamma }\right)\rangle$ in ℜ. Then, score and accuracy values are defined as*

*(1) $\tilde{S}\left(\varepsilon_{\rho }\right)=\frac{1}{3}\left(2+P_{\xi_{\rho }}-I_{\xi_{\rho }}-N_{\xi_{\rho }}\right)\in \left[0,1\right]$*

*(2) $\tilde{A}\left(\varepsilon_{\rho }\right)=\left(P_{\xi_{\rho }}-N_{\xi_{\rho }}\right)\in \left[0,1\right].$*

The score and accuracy values defined above suggest which SFN is greater than other SFNs. The comparison technique is defined in the next definition.

**Definition** **10.** *For any SFNs, $\varepsilon_{\rho }=\langle P_{\xi_{\rho }}\left({\overset{ˇ}{r}}_{\gamma }\right),I_{\xi_{\rho }}\left({\overset{ˇ}{r}}_{\gamma }\right),N_{\xi_{\rho }}\left({\overset{ˇ}{r}}_{\gamma }\right)\rangle$$\left(\rho =1,2\right)$ in ℜ. Then, the comparison technique is proposed as*

*(1) If $\tilde{S}\left(\varepsilon_{1}\right)<\tilde{S}\left(\varepsilon_{2}\right),$ then $\varepsilon_{1}<\varepsilon_{2},$*

*(2) If $\tilde{S}\left(\varepsilon_{1}\right)>\tilde{S}\left(\varepsilon_{2}\right),$ then $\varepsilon_{1}>\varepsilon_{2},$*

*(3) If $\tilde{S}\left(\varepsilon_{1}\right)=\tilde{S}\left(\varepsilon_{2}\right),$ then*

*(a) $\tilde{A}\left(\varepsilon_{1}\right)<\tilde{A}\left(\varepsilon_{2}\right),$ then $\varepsilon_{1}<\varepsilon_{2},$*

*(b) $\tilde{A}\left(\varepsilon_{1}\right)>\tilde{A}\left(\varepsilon_{2}\right),$ then $\varepsilon_{1}>\varepsilon_{2},$*

*(c) $\tilde{A}\left(\varepsilon_{1}\right)=\tilde{A}\left(\varepsilon_{2}\right),$ then $\varepsilon_{1}\approx \varepsilon_{2}.$*

Ashraf and Abdullah [40] proposed aggregation operators for SFNs based on different norms:

**Definition** **11.** *For any collection of SFNs, $\varepsilon_{\rho }=\langle P_{\xi_{\rho }}\left({\overset{ˇ}{r}}_{\gamma }\right),I_{\xi_{\rho }}\left({\overset{ˇ}{r}}_{\gamma }\right),N_{\xi_{\rho }}\left({\overset{ˇ}{r}}_{\gamma }\right)\rangle$$\left(\rho =1,2,\ldots ,n\right)$ in ℜ. The structure of the spherical weighted averaging (SFWA) operator is*

$$SFWA\left(\varepsilon_{1},\varepsilon_{2},\ldots ,\varepsilon_{n}\right)=\sum_{\rho =1}^{n}\beta_{\rho }\varepsilon_{\rho },$$

*where $\beta_{\rho }\left(\rho =1,2,\ldots ,n\right)$ are weight vectors with $\beta_{\rho }\geq 0$ and $\sum_{\rho =1}^{n}\beta_{\rho }=1.$*

**Definition** **12.** *For any collection of SFNs, $\varepsilon_{\rho }=\langle P_{\xi_{\rho }}\left({\overset{ˇ}{r}}_{\gamma }\right),I_{\xi_{\rho }}\left({\overset{ˇ}{r}}_{\gamma }\right),N_{\xi_{\rho }}\left({\overset{ˇ}{r}}_{\gamma }\right)\rangle$$\left(\rho =1,2,\ldots ,n\right)$ in ℜ. The structure of the spherical order weighted averaging (SFOWA) operator is*

$$SFOWA\left(\varepsilon_{1},\varepsilon_{2},\ldots ,\varepsilon_{n}\right)=\sum_{\rho =1}^{n}\beta_{\rho }\varepsilon_{\eta (\rho )},$$

*where $\beta_{\rho }\left(\rho =1,2,\ldots ,n\right)$ are weight vectors with $\beta_{\rho }\geq 0$, $\sum_{\rho =1}^{n}\beta_{\rho }=1$ and the ρth biggest weighted value is $\varepsilon_{\eta (\rho )}$ consequently by total order $\varepsilon_{\eta (1)}\geq \varepsilon_{\eta (2)}\geq \ldots \geq \varepsilon_{\eta (n)}.$*

**Definition** **13.** *For any collection of SFNs, $\varepsilon_{\rho }=\langle P_{\xi_{\rho }}\left({\overset{ˇ}{r}}_{\gamma }\right),I_{\xi_{\rho }}\left({\overset{ˇ}{r}}_{\gamma }\right),N_{\xi_{\rho }}\left({\overset{ˇ}{r}}_{\gamma }\right)\rangle$$\left(\rho =1,2,\ldots ,n\right)$ in ℜ. The structure of the spherical hybrid weighted averaging (SFHWA) operator is*

$$SFHWA\left(\varepsilon_{1},\varepsilon_{2},\ldots ,\varepsilon_{n}\right)=\sum_{\rho =1}^{n}\beta_{\rho }\varepsilon_{\eta (\rho )}^{*},$$

where $\beta_{\rho }\left(\rho =1,2,\ldots ,n\right)$ are weight vectors with $\beta_{\rho }\geq 0$, $\sum_{\rho =1}^{n}\beta_{\rho }=1$ and the ρth biggest weighted value is $\varepsilon_{\eta (\rho )}^{*}\left(\varepsilon_{\eta (\rho )}^{*}=n\beta_{\rho }\varepsilon_{\eta (\rho )},\;\rho \in N\right)$ consequently by total order $\varepsilon_{\eta (1)}^{*}\geq \varepsilon_{\eta (2)}^{*}\geq \ldots \geq \varepsilon_{\eta (n)}^{*}.$ In addition, the associated weights are $\omega =(\omega_{1},\omega_{2},\ldots ,\omega_{n})$ with $\omega_{\rho }\geq 0$, $\Sigma_{\rho =1}^{n}\omega_{\rho }=1.$

**Definition** **14.** *For any collection of SFNs, $\varepsilon_{\rho }=\langle P_{\xi_{\rho }}\left({\overset{ˇ}{r}}_{\gamma }\right),I_{\xi_{\rho }}\left({\overset{ˇ}{r}}_{\gamma }\right),N_{\xi_{\rho }}\left({\overset{ˇ}{r}}_{\gamma }\right)\rangle$$\left(\rho =1,2,\ldots ,n\right)$ in ℜ. The structure of spherical weighted geometric (SFWG) operator is*

$$SFWG\left(\varepsilon_{1},\varepsilon_{2},\ldots ,\varepsilon_{n}\right)=\prod_{\rho =1}^{n}{\left(\varepsilon_{\rho }\right)}^{\beta_{\rho }},$$

*where $\beta_{\rho }\left(\rho =1,2,\ldots ,n\right)$ are weight vectors with $\beta_{\rho }\geq 0$ and $\sum_{\rho =1}^{n}\beta_{\rho }=1.$*

**Definition** **15.** *For any collection of SFNs, $\varepsilon_{\rho }=\langle P_{\xi_{\rho }}\left({\overset{ˇ}{r}}_{\gamma }\right),I_{\xi_{\rho }}\left({\overset{ˇ}{r}}_{\gamma }\right),N_{\xi_{\rho }}\left({\overset{ˇ}{r}}_{\gamma }\right)\rangle$$\left(\rho =1,2,\ldots ,n\right)$ in ℜ. The structure of the spherical order weighted geometric (SFOWG) operator is*

$$SFOWG\left(\varepsilon_{1},\varepsilon_{2},\ldots ,\varepsilon_{n}\right)=\prod_{\rho =1}^{n}{\left(\varepsilon_{\eta (\rho )}\right)}^{\beta_{\rho }},$$

*where $\beta_{\rho }\left(\rho =1,2,\ldots ,n\right)$ are weight vectors with $\beta_{\rho }\geq 0$, $\sum_{\rho =1}^{n}\beta_{\rho }=1$ and the ρth biggest weighted value is $\varepsilon_{\eta (\rho )}$ consequently by total order $\varepsilon_{\eta (1)}\geq \varepsilon_{\eta (2)}\geq \ldots \geq \varepsilon_{\eta (n)}.$*

**Definition** **16.** *For any collection of SFNs, $\varepsilon_{\rho }=\langle P_{\xi_{\rho }}\left({\overset{ˇ}{r}}_{\gamma }\right),I_{\xi_{\rho }}\left({\overset{ˇ}{r}}_{\gamma }\right),N_{\xi_{\rho }}\left({\overset{ˇ}{r}}_{\gamma }\right)\rangle$$\left(\rho =1,2,\ldots ,n\right)$ in ℜ. The structure of spherical hybrid weighted geometric (SFHWG) operator is*

$$SFHWG\left(\varepsilon_{1},\varepsilon_{2},\ldots ,\varepsilon_{n}\right)=\prod_{\rho =1}^{n}{\left(\varepsilon_{\eta (\rho )}^{*}\right)}^{\beta_{\rho }},$$

where $\beta_{\rho }\left(\rho =1,2,\ldots ,n\right)$ are weight vectors with $\beta_{\rho }\geq 0$, $\sum_{\rho =1}^{n}\beta_{\rho }=1$ and the ρth biggest weighted value is $\varepsilon_{\eta (\rho )}^{*}\left(\varepsilon_{\eta (\rho )}^{*}=n\beta_{\rho }\varepsilon_{\eta (\rho )},\;\rho \in N\right)$ consequently by total order $\varepsilon_{\eta (1)}^{*}\geq \varepsilon_{\eta (2)}^{*}\geq \ldots \geq \varepsilon_{\eta (n)}^{*}.$ In addition, associated weights are $\omega =(\omega_{1},\omega_{2},\ldots ,\omega_{n})$ with $\omega_{\rho }\geq 0$, $\Sigma_{\rho =1}^{n}\omega_{\rho }=1.$

## 3. Entropy

Basically, we familiarize the concept of entropy, when probability measures the discrimination of criteria being imposed on multi-attribute decision-making problems. Non-probabilistic entropy firstly approximated by De Luca and Termini [46] also presented some necessities to find intuitive comprehension of the degree of fuzziness. Many researchers are getting interest in this field and have done a lot of work such as Scmidt and Kacprzyk [47] proposing some axioms for distance between intuitionistic fuzzy sets and non-probabilistic entropy measure for them. In this section, we recall the concept of Shannon entropy, fuzzy entropy, entropy for Pythagorean fyzzy numbers and propose the entropy for spherical fuzzy numbers.

**Definition** **17** ([48])**.** *Let $\delta \left(\eta_{\rho }\right)$$\rho \in \left\{1,2,\ldots ,n\right\}$ be the set of n-complete probability distributions. Shannon entropy for $\delta \left(\eta_{\rho }\right)$$\rho \in \left\{1,2,\ldots ,n\right\}$ probability distribution is defined as*

$$E_{s}\left(\delta \right)=-\sum_{\rho =1}^{n}\delta \left(\eta_{\rho }\right)\log\delta \left(\eta_{\rho }\right).$$

**Definition** **18** ([49])**.** *Let F be any fuzzy set in ℜ, fuzzy entropy for the set F, we mean a structure*

$$F\left(\delta \right)=-\frac{1}{n}\sum_{\rho =1}^{n}\left[P\left(\eta_{\rho }\right)\log P\left(\eta_{\rho }\right)+\left(1-P\left(\eta_{\rho }\right)\right)\log\left(1-P\left(\eta_{\rho }\right)\right)\right].$$

**Definition** **19** ([49])**.** *Let F be any fuzzy set in ℜ, and, for Pythagorean fuzzy entropy for the set F, we mean a structure*

$$Py_{q}=\frac{1+\frac{1}{n}\sum_{\rho =1}^{n}\left(P_{i}\log\left(P_{i}\right)+N_{i}\log\left(N_{i}\right)\right)}{\sum_{q=1}^{n}\left(1+\frac{1}{n}\sum_{\rho =1}^{n}P_{i}\log\left(P_{i}\right)+N_{i}\log\left(N_{i}\right)\right)}.$$

**Definition** **20.** *Let F be any fuzzy set in ℜ, spherical fuzzy entropy for the set F, and we mean a structure*

$$\gamma_{q}=\frac{1+\frac{1}{n}\sum_{\rho =1}^{n}\left(P_{i}\log\left(P_{i}\right)+I_{i}\log\left(I_{i}\right)+N_{i}\log\left(N_{i}\right)\right)}{\sum_{q=1}^{n}\left(1+\frac{1}{n}\sum_{\rho =1}^{n}P_{i}\log\left(P_{i}\right)+I_{i}\log\left(I_{i}\right)+N_{i}\log\left(N_{i}\right)\right)}.$$

## 4. Spherical Fuzzy Logarithmic Operational Laws

Motivated by the novel concept of spherical fuzzy set, we introduced some novel logarithmic operational laws for SFNs. As real number system $\ell og_{\sigma }0$ is meaningless and $\ell og_{\sigma }1$ is not defined therefore, in our study, we take nonempty spherical fuzzy sets and σ≠1, where σ is any real number.

**Definition** **21.** *For any SFN, $\varepsilon_{\rho }=\langle P_{\xi_{\rho }}\left({\overset{ˇ}{r}}_{\gamma }\right),I_{\xi_{\rho }}\left({\overset{ˇ}{r}}_{\gamma }\right),N_{\xi_{\rho }}\left({\overset{ˇ}{r}}_{\gamma }\right)\rangle$ in ℜ. The logarithmic spherical fuzzy number is defined as*

$$\ell og_{\sigma }\varepsilon_{\rho }=\left\{\langle \sqrt{1-{\left(\ell og_{\sigma }P_{\xi_{\rho }}\left({\overset{ˇ}{r}}_{\gamma }\right)\right)}^{2}},\ell og_{\sigma }\left(\sqrt{1-I_{\xi_{\rho }}^{2}\left({\overset{ˇ}{r}}_{\gamma }\right)}\right),\ell og_{\sigma }\left(\sqrt{1-N_{\xi_{\rho }}^{2}\left({\overset{ˇ}{r}}_{\gamma }\right)}\right)\rangle |{\overset{ˇ}{r}}_{\gamma }\in ℜ\right\}$$

*in which $P_{\sigma }:ℜ\to \Theta ,$$I_{\sigma }:ℜ\to \Theta$ and $N_{\sigma }:ℜ\to \Theta$ indicate that the positive, neutral and negative grades in ℜ, $\Theta =\left[0,1\right]$ are the unit intervals. In addition, the following condition satisfies by $P_{\sigma },I_{\sigma }$ and $N_{\sigma }$ is $0\leq P_{\sigma }^{2}\left({\overset{ˇ}{r}}_{\gamma }\right)+I_{\sigma }^{2}\left({\overset{ˇ}{r}}_{\gamma }\right)+N_{\sigma }^{2}\left({\overset{ˇ}{r}}_{\gamma }\right)\leq 1$ for all ${\overset{ˇ}{r}}_{\gamma }\in ℜ.$ Therefore, the membership grade is*

$$\sqrt{1-{\left(\ell og_{\sigma }P_{\xi_{\rho }}\left({\overset{ˇ}{r}}_{\gamma }\right)\right)}^{2}}:ℜ\to \Theta ,\;\mathrm{such}\;\mathrm{that}\;0\leq \sqrt{1-{\left(\ell og_{\sigma }P_{\xi_{\rho }}\left({\overset{ˇ}{r}}_{\gamma }\right)\right)}^{2}}\leq 1,\forall {\overset{ˇ}{r}}_{\gamma }\in ℜ,$$

*the neutral grade is*

$$\ell og_{\sigma }\left(\sqrt{1-I_{\xi_{\rho }}^{2}\left({\overset{ˇ}{r}}_{\gamma }\right)}\right):ℜ\to \Theta ,\;\mathrm{such}\;\mathrm{that}\;0\leq \ell og_{\sigma }\left(\sqrt{1-I_{\xi_{\rho }}^{2}\left({\overset{ˇ}{r}}_{\gamma }\right)}\right)\leq 1,\forall {\overset{ˇ}{r}}_{\gamma }\in ℜ,$$

*and the negative grade is*

$$\ell og_{\sigma }\left(\sqrt{1-N_{\xi_{\rho }}^{2}\left({\overset{ˇ}{r}}_{\gamma }\right)}\right):ℜ\to \Theta ,\mathrm{such}\;\mathrm{that}\;0\leq \ell og_{\sigma }\left(\sqrt{1-N_{\xi_{\rho }}^{2}\left({\overset{ˇ}{r}}_{\gamma }\right)}\right)\leq 1,\forall {\overset{ˇ}{r}}_{\gamma }\in ℜ.$$

*Therefore,*

$$\begin{array}{ccc} \ell og_{\sigma }\varepsilon_{\rho } & = & \left\{\langle \sqrt{1-{\left(\ell og_{\sigma }P_{\xi_{\rho }}\left({\overset{ˇ}{r}}_{\gamma }\right)\right)}^{2}},\ell og_{\sigma }\left(\sqrt{1-I_{\xi_{\rho }}^{2}\left({\overset{ˇ}{r}}_{\gamma }\right)}\right),\ell og_{\sigma }\left(\sqrt{1-N_{\xi_{\rho }}^{2}\left({\overset{ˇ}{r}}_{\gamma }\right)}\right)\rangle |{\overset{ˇ}{r}}_{\gamma }\in ℜ\right\}\\ 0 & < & \sigma \leq \min\left\{P_{\xi_{\rho }},\sqrt{1-I_{\xi_{\rho }}^{2}},\sqrt{1-N_{\xi_{\rho }}^{2}}\right\}\leq 1,\;\sigma \neq 1 \end{array}$$

*is SPN.*

**Definition** **22.** *For any SFN, $\varepsilon_{\rho }=\langle P_{\xi_{\rho }}\left({\overset{ˇ}{r}}_{\gamma }\right),I_{\xi_{\rho }}\left({\overset{ˇ}{r}}_{\gamma }\right),N_{\xi_{\rho }}\left({\overset{ˇ}{r}}_{\gamma }\right)\rangle$ in ℜ. If*

$$\ell og_{\sigma }\varepsilon_{\rho }=\left\{\begin{array}{cc} \left(\begin{array}{c} \sqrt{1-{\left(\ell og_{\sigma }P_{\xi_{\rho }}\left({\overset{ˇ}{r}}_{\gamma }\right)\right)}^{2}},\\ \ell og_{\sigma }\left(\sqrt{1-I_{\xi_{\rho }}^{2}\left({\overset{ˇ}{r}}_{\gamma }\right)}\right),\\ \ell og_{\sigma }\left(\sqrt{1-N_{\xi_{\rho }}^{2}\left({\overset{ˇ}{r}}_{\gamma }\right)}\right) \end{array}\right) & 0<\sigma \leq \min\left\{P_{\xi_{\rho }},\sqrt{1-I_{\xi_{\rho }}^{2}},\sqrt{1-N_{\xi_{\rho }}^{2}}\right\}<1,\;\\ \left(\begin{array}{c} \sqrt{1-{\left(\ell og_{\frac{1}{\sigma }}P_{\xi_{\rho }}\left({\overset{ˇ}{r}}_{\gamma }\right)\right)}^{2}},\\ \ell og_{\frac{1}{\sigma }}\left(\sqrt{1-I_{\xi_{\rho }}^{2}\left({\overset{ˇ}{r}}_{\gamma }\right)}\right),\\ \ell og_{\frac{1}{\sigma }}\left(\sqrt{1-N_{\xi_{\rho }}^{2}\left({\overset{ˇ}{r}}_{\gamma }\right)}\right) \end{array}\right) & \begin{array}{c} 0<\frac{1}{\sigma }\leq \min\left\{P_{\xi_{\rho }},\sqrt{1-I_{\xi_{\rho }}^{2}},\sqrt{1-N_{\xi_{\rho }}^{2}}\right\}<1,\;\\ \sigma \neq 1, \end{array} \end{array}\right.$$

*then the function $\ell og_{\sigma }\varepsilon_{\rho }$ is known to be a logarithmic operator for a spherical fuzzy set, and its value is called logarithmic SFN (L-SFN). Here, we take $\ell og_{\sigma }0=0,\sigma >0,\sigma \neq 1.$*

**Theorem** **1.** *For any SFN, $\varepsilon_{\rho }=\langle P_{\xi_{\rho }}\left({\overset{ˇ}{r}}_{\gamma }\right),I_{\xi_{\rho }}\left({\overset{ˇ}{r}}_{\gamma }\right),N_{\xi_{\rho }}\left({\overset{ˇ}{r}}_{\gamma }\right)\rangle$ in ℜ, then $\ell og_{\sigma }\varepsilon_{\rho }$ is also a spherical fuzzy number.*

**Proof.**  Since any SFN $\varepsilon_{\rho }=\langle P_{\xi_{\rho }}\left({\overset{ˇ}{r}}_{\gamma }\right),I_{\xi_{\rho }}\left({\overset{ˇ}{r}}_{\gamma }\right),N_{\xi_{\rho }}\left({\overset{ˇ}{r}}_{\gamma }\right)\rangle$ in *ℜ*, which means that $P_{\sigma }:ℜ\to \Theta ,$ $I_{\sigma }:ℜ\to \Theta$ and $N_{\sigma }:ℜ\to \Theta$ indicate that the positive, neutral and negative grades in *ℜ*, $\Theta =\left[0,1\right]$ are the unit intervals. In addition, the following condition satisfied by $P_{\sigma },I_{\sigma }$ and $N_{\sigma }$ is $0\leq P_{\sigma }^{2}\left({\overset{ˇ}{r}}_{\gamma }\right)+I_{\sigma }^{2}\left({\overset{ˇ}{r}}_{\gamma }\right)+N_{\sigma }^{2}\left({\overset{ˇ}{r}}_{\gamma }\right)\leq 1.$ The following two cases happen.

Case-1 When $0<\sigma \leq \min\left\{P_{\xi_{\rho }},\sqrt{1-I_{\xi_{\rho }}^{2}},\sqrt{1-N_{\xi_{\rho }}^{2}}\right\}<1,$ σ≠1 and since $\ell og_{\sigma }\varepsilon_{\rho }$ is a decreasing function w.r.t σ. Thus, $0\leq \ell og_{\sigma }P_{\xi_{\rho }},\ell og_{\sigma }\left(\sqrt{1-I_{\xi_{\rho }}^{2}}\right),\ell og_{\sigma }\left(\sqrt{1-N_{\xi_{\rho }}^{2}}\right)\leq 1$ and hence $0\leq \sqrt{1-{\left(\ell og_{\sigma }P_{\xi_{\rho }}\left({\overset{ˇ}{r}}_{\gamma }\right)\right)}^{2}}\leq 1,$ $0\leq \ell og_{\sigma }\left(\sqrt{1-I_{\xi_{\rho }}^{2}\left({\overset{ˇ}{r}}_{\gamma }\right)}\right)\leq 1$, $0\leq \ell og_{\sigma }\left(\sqrt{1-N_{\xi_{\rho }}^{2}\left({\overset{ˇ}{r}}_{\gamma }\right)}\right)\leq 1$ and $0\leq \sqrt{1-{\left(\ell og_{\sigma }P_{\xi_{\rho }}\left({\overset{ˇ}{r}}_{\gamma }\right)\right)}^{2}}+\ell og_{\sigma }\left(\sqrt{1-I_{\xi_{\rho }}^{2}\left({\overset{ˇ}{r}}_{\gamma }\right)}\right)+\ell og_{\sigma }\left(\sqrt{1-N_{\xi_{\rho }}^{2}\left({\overset{ˇ}{r}}_{\gamma }\right)}\right)\leq 1.$ Therefore, $\ell og_{\sigma }\varepsilon_{\rho }$ is SFN.

Case-2 When σ>1, $0<\frac{1}{\sigma }<1$ and $\frac{1}{\sigma }\leq \min\left\{P_{\xi_{\rho }},\sqrt{1-I_{\xi_{\rho }}^{2}},\sqrt{1-N_{\xi_{\rho }}^{2}}\right\};$ similar to the above, we can find that $\ell og_{\sigma }\varepsilon_{\rho }$ is SFN. Thus, the procedure is eliminated here. □

**Example** **1.** *Suppose that, for any SFN, $\varepsilon_{\rho }=\langle 0.8,0.5,0.3\rangle$ in ℜ with σ=0.4, then*

$$\begin{array}{ccc} \ell og_{\sigma }\varepsilon_{\rho } & = & \left(\sqrt{1-{\left(\ell og_{0.4}\left(0.8\right)\right)}^{2}},\ell og_{0.4}\left(\sqrt{1-{\left(0.5\right)}^{2}}\right),\ell og_{0.4}\left(\sqrt{1-{\left(0.3\right)}^{2}}\right)\right)\\ & = & \left(0.969,0.156,0.051\right). \end{array}$$

*In addition, if σ=8, then it follows:*

$$\begin{array}{ccc} \ell og_{\frac{1}{\sigma }}\varepsilon_{\rho } & = & \left(\sqrt{1-{\left(\ell og_{\frac{1}{8}}\left(0.8\right)\right)}^{2}},\ell og_{\frac{1}{8}}\left(\sqrt{1-{\left(0.5\right)}^{2}}\right),\ell og_{\frac{1}{8}}\left(\sqrt{1-{\left(0.3\right)}^{2}}\right)\right)\\ & = & \left(0.994,0.069,0.022\right). \end{array}$$

Now, we give some discussion on the basic properties of the L-SFN.

**Theorem** **2.** *For any SFN, $\varepsilon_{\rho }=\langle P_{\xi_{\rho }}\left({\overset{ˇ}{r}}_{\gamma }\right),I_{\xi_{\rho }}\left({\overset{ˇ}{r}}_{\gamma }\right),N_{\xi_{\rho }}\left({\overset{ˇ}{r}}_{\gamma }\right)\rangle$ in ℜ. If $0<\sigma \leq \min\left\{P_{\xi_{\rho }},\sqrt{1-I_{\xi_{\rho }}^{2}},\sqrt{1-N_{\xi_{\rho }}^{2}}\right\}<1,$σ≠1 then*

*(1) $\sigma^{\ell og_{\sigma }\varepsilon_{\rho }}=\varepsilon_{\rho };$*

*(2) $\ell og_{\sigma }\sigma^{\varepsilon_{\rho }}=\varepsilon_{\rho }.$*

**Proof.**  (1) According to Definitions 7 and 22, we obtain

$$\begin{array}{ccc} \sigma^{\ell og_{\sigma }\varepsilon_{\rho }} & = & \left(\sigma^{\sqrt{1-{\left(\sqrt{1-{\left(\ell og_{\sigma }\rho_{\xi_{\rho }}\right)}^{2}}\right)}^{2}}},\sqrt{1-\sigma^{2\ell og_{\sigma }\sqrt{1-I_{\xi_{\rho }}^{2}}}},\sqrt{1-\sigma^{2\ell og_{\sigma }\sqrt{1-N_{\xi_{\rho }}^{2}}}}\right)\\ & = & \left(\sigma^{\sqrt{1-\left(1-{\left(\ell og_{\sigma }\rho_{\xi_{\rho }}\right)}^{2}\right)}},\sqrt{1-\left(1-I_{\xi_{\rho }}^{2}\right)},\sqrt{1-\left(1-N_{\xi_{\rho }}^{2}\right)}\right)\\ & = & \left(\sigma^{\ell og_{\sigma }\rho_{\xi_{\rho }}},I_{\xi_{\rho }},N_{\xi_{\rho }}\right)\\ & = & \left(\rho_{\xi_{\rho }},I_{\xi_{\rho }},N_{\xi_{\rho }}\right)=\varepsilon_{\rho }. \end{array}$$

(2) According to Definition 22, we obtain

$$\begin{array}{ccc} \ell og_{\sigma }\sigma^{\varepsilon_{\rho }} & = & \ell og_{\sigma }\left(\sigma^{\sqrt{1-\rho_{\xi_{\rho }}^{2}}},\sqrt{1-\sigma^{2I_{\xi_{\rho }}}},\sqrt{1-\sigma^{2N_{\xi_{\rho }}}}\right)\\ & = & \left(\begin{array}{c} \sqrt{1-{\left(\ell og_{\sigma }\sigma^{\sqrt{1-\rho_{\xi_{\rho }}^{2}}}\right)}^{2}},\ell og_{\sigma }\left(\sqrt{1-{\left(\sqrt{1-\sigma^{2I_{\xi_{\rho }}}}\right)}^{2}}\right),\\ \ell og_{\sigma }\left(\sqrt{1-{\left(\sqrt{1-\sigma^{2N_{\xi_{\rho }}}}\right)}^{2}}\right) \end{array}\right)\\ & = & \left(\begin{array}{c} \sqrt{1-\left(1-\rho_{\xi_{\rho }}^{2}\right)},\ell og_{\sigma }\left(\sqrt{1-\left(1-\sigma^{2I_{\xi_{\rho }}}\right)}\right),\\ \ell og_{\sigma }\left(\sqrt{1-\left(1-\sigma^{2N_{\xi_{\rho }}}\right)}\right) \end{array}\right)\\ & = & \left(\rho_{\xi_{\rho }},I_{\xi_{\rho }},N_{\xi_{\rho }}\right)=\varepsilon_{\rho }. \end{array}$$

□

**Definition** **23.** *For any two L-SFNs, $\ell og_{\sigma }\varepsilon_{\rho }=\left(\begin{array}{c} \sqrt{1-{\left(\ell og_{\sigma }P_{\xi_{\rho }}\left({\overset{ˇ}{r}}_{\gamma }\right)\right)}^{2}},\\ \ell og_{\sigma }\left(\sqrt{1-I_{\xi_{\rho }}^{2}\left({\overset{ˇ}{r}}_{\gamma }\right)}\right),\\ \ell og_{\sigma }\left(\sqrt{1-N_{\xi_{\rho }}^{2}\left({\overset{ˇ}{r}}_{\gamma }\right)}\right) \end{array}\right)$ and $\ell og_{\sigma }\varepsilon_{q}=\left(\begin{array}{c} \sqrt{1-{\left(\ell og_{\sigma }P_{\xi_{q}}\left({\overset{ˇ}{r}}_{\gamma }\right)\right)}^{2}},\\ \ell og_{\sigma }\left(\sqrt{1-I_{\xi_{q}}^{2}\left({\overset{ˇ}{r}}_{\gamma }\right)}\right),\\ \ell og_{\sigma }\left(\sqrt{1-N_{\xi_{q}}^{2}\left({\overset{ˇ}{r}}_{\gamma }\right)}\right) \end{array}\right)$ in ℜ and β≥0, then the logarithmic operations of L-SFNs are proposed:*

*(1) $\ell og_{\sigma }\varepsilon_{\rho }⊕\ell og_{\sigma }\varepsilon_{q}=\left\{\begin{array}{c} \sqrt{1-{\left(\ell og_{\sigma }P_{\xi_{\rho }}\left({\overset{ˇ}{r}}_{\gamma }\right)\right)}^{2}\cdot {\left(\ell og_{\sigma }P_{\xi_{q}}\left({\overset{ˇ}{r}}_{\gamma }\right)\right)}^{2}},\\ \ell og_{\sigma }\left(\sqrt{1-I_{\xi_{\rho }}^{2}\left({\overset{ˇ}{r}}_{\gamma }\right)}\right)\cdot \ell og_{\sigma }\left(\sqrt{1-I_{\xi_{q}}^{2}\left({\overset{ˇ}{r}}_{\gamma }\right)}\right),\\ \ell og_{\sigma }\left(\sqrt{1-N_{\xi_{\rho }}^{2}\left({\overset{ˇ}{r}}_{\gamma }\right)}\right)\cdot \ell og_{\sigma }\left(\sqrt{1-N_{\xi_{q}}^{2}\left({\overset{ˇ}{r}}_{\gamma }\right)}\right) \end{array}\right\};$*

*(2) $\beta \cdot \ell og_{\sigma }\varepsilon_{\rho }=\left\{\begin{array}{c} \sqrt{1-{\left(\ell og_{\sigma }P_{\xi_{\rho }}\left({\overset{ˇ}{r}}_{\gamma }\right)\right)}^{2\beta }},\\ {\left(\ell og_{\sigma }\left(\sqrt{1-I_{\xi_{\rho }}^{2}\left({\overset{ˇ}{r}}_{\gamma }\right)}\right)\right)}^{\beta },\\ {\left(\ell og_{\sigma }\left(\sqrt{1-N_{\xi_{\rho }}^{2}\left({\overset{ˇ}{r}}_{\gamma }\right)}\right)\right)}^{\beta } \end{array}\right\};$*

*(3) $\ell og_{\sigma }\varepsilon_{\rho }⊗\ell og_{\sigma }\varepsilon_{q}=\left\{\begin{array}{c} \sqrt{1-{\left(\ell og_{\sigma }P_{\xi_{\rho }}\left({\overset{ˇ}{r}}_{\gamma }\right)\right)}^{2}}\cdot \sqrt{1-{\left(\ell og_{\sigma }P_{\xi_{q}}\left({\overset{ˇ}{r}}_{\gamma }\right)\right)}^{2}},\\ \sqrt{1-{\left(1-\ell og_{\sigma }\left(\sqrt{1-I_{\xi_{\rho }}^{2}\left({\overset{ˇ}{r}}_{\gamma }\right)}\right)\right)}^{2}\cdot {\left(1-\ell og_{\sigma }\left(\sqrt{1-I_{\xi_{q}}^{2}\left({\overset{ˇ}{r}}_{\gamma }\right)}\right)\right)}^{2}},\\ \sqrt{1-{\left(1-\ell og_{\sigma }\left(\sqrt{1-N_{\xi_{\rho }}^{2}\left({\overset{ˇ}{r}}_{\gamma }\right)}\right)\right)}^{2}\cdot {\left(1-\ell og_{\sigma }\left(\sqrt{1-N_{\xi_{q}}^{2}\left({\overset{ˇ}{r}}_{\gamma }\right)}\right)\right)}^{2}} \end{array}\right\};$*

*(4) ${\left(\ell og_{\sigma }\varepsilon_{\rho }\right)}^{\beta }=\left\{\begin{array}{c} {\left(\sqrt{1-{\left(\ell og_{\sigma }P_{\xi_{\rho }}\left({\overset{ˇ}{r}}_{\gamma }\right)\right)}^{2}}\right)}^{\beta },\\ \sqrt{1-{\left(1-{\left(\ell og_{\sigma }\left(\sqrt{1-I_{\xi_{\rho }}^{2}\left({\overset{ˇ}{r}}_{\gamma }\right)}\right)\right)}^{2}\right)}^{\beta }},\\ \sqrt{1-{\left(1-{\left(\ell og_{\sigma }\left(\sqrt{1-I_{\xi_{\rho }}^{2}\left({\overset{ˇ}{r}}_{\gamma }\right)}\right)\right)}^{2}\right)}^{\beta }} \end{array}\right\}.$*

**Theorem** **3.** *For any two L-SFNs, $\ell og_{\sigma }\varepsilon_{\rho }=\left(\begin{array}{c} \sqrt{1-{\left(\ell og_{\sigma }P_{\xi_{\rho }}\left({\overset{ˇ}{r}}_{\gamma }\right)\right)}^{2}},\\ \ell og_{\sigma }\left(\sqrt{1-I_{\xi_{\rho }}^{2}\left({\overset{ˇ}{r}}_{\gamma }\right)}\right),\\ \ell og_{\sigma }\left(\sqrt{1-N_{\xi_{\rho }}^{2}\left({\overset{ˇ}{r}}_{\gamma }\right)}\right) \end{array}\right)$$\left(\rho =1,2\right)$ in ℜ, with $0<\sigma \leq \min\left\{P_{\xi_{\rho }},\sqrt{1-I_{\xi_{\rho }}^{2}},\sqrt{1-N_{\xi_{\rho }}^{2}}\right\}<1,$σ≠1. Then,*

*(1) $\ell og_{\sigma }\varepsilon_{1}⊕\ell og_{\sigma }\varepsilon_{2}=\ell og_{\sigma }\varepsilon_{2}⊕\ell og_{\sigma }\varepsilon_{1},$*

*(2)$\ell og_{\sigma }\varepsilon_{1}⊗\ell og_{\sigma }\varepsilon_{2}=\ell og_{\sigma }\varepsilon_{2}⊗\ell og_{\sigma }\varepsilon_{1}.$*

**Proof.**  This is straightforward from Definition 23, so the procedure is eliminated here. □

**Theorem** **4.** *For any two L-SFNs, $\ell og_{\sigma }\varepsilon_{\rho }=\left(\begin{array}{c} \sqrt{1-{\left(\ell og_{\sigma }P_{\xi_{\rho }}\left({\overset{ˇ}{r}}_{\gamma }\right)\right)}^{2}},\\ \ell og_{\sigma }\left(\sqrt{1-I_{\xi_{\rho }}^{2}\left({\overset{ˇ}{r}}_{\gamma }\right)}\right),\\ \ell og_{\sigma }\left(\sqrt{1-N_{\xi_{\rho }}^{2}\left({\overset{ˇ}{r}}_{\gamma }\right)}\right) \end{array}\right)$$\left(\rho =1,2,3\right)$ in ℜ, with $0<\sigma \leq \min\left\{P_{\xi_{\rho }},\sqrt{1-I_{\xi_{\rho }}^{2}},\sqrt{1-N_{\xi_{\rho }}^{2}}\right\}<1,$σ≠1. Then,*

*(1) $\left(\ell og_{\sigma }\varepsilon_{1}⊕\ell og_{\sigma }\varepsilon_{2}\right)⊕\ell og_{\sigma }\varepsilon_{3}=\ell og_{\sigma }\varepsilon_{1}⊕\left(\ell og_{\sigma }\varepsilon_{2}⊕\ell og_{\sigma }\varepsilon_{3}\right),$*

*(2)$\left(\ell og_{\sigma }\varepsilon_{1}⊗\ell og_{\sigma }\varepsilon_{2}\right)⊗\ell og_{\sigma }\varepsilon_{3}=\ell og_{\sigma }\varepsilon_{1}⊗\left(\ell og_{\sigma }\varepsilon_{2}⊗\ell og_{\sigma }\varepsilon_{3}\right).$*

**Proof.**  This is straightforward from Definition 23, so the procedure is eliminated here. □

**Theorem** **5.** *For any two L-SFNs, $\ell og_{\sigma }\varepsilon_{\rho }=\left(\begin{array}{c} \sqrt{1-{\left(\ell og_{\sigma }P_{\xi_{\rho }}\left({\overset{ˇ}{r}}_{\gamma }\right)\right)}^{2}},\\ \ell og_{\sigma }\left(\sqrt{1-I_{\xi_{\rho }}^{2}\left({\overset{ˇ}{r}}_{\gamma }\right)}\right),\\ \ell og_{\sigma }\left(\sqrt{1-N_{\xi_{\rho }}^{2}\left({\overset{ˇ}{r}}_{\gamma }\right)}\right) \end{array}\right)$$\left(\rho =1,2\right)$ in ℜ, with $0<\sigma \leq \min\left\{P_{\xi_{\rho }},\sqrt{1-I_{\xi_{\rho }}^{2}},\sqrt{1-N_{\xi_{\rho }}^{2}}\right\}<1,$σ≠1,$\beta ,\beta_{1},\beta_{2}>0$ be any real numbers. Then,*

*(1) $\beta \left(\ell og_{\sigma }\varepsilon_{1}⊕\ell og_{\sigma }\varepsilon_{2}\right)=\beta \ell og_{\sigma }\varepsilon_{1}⊕\beta \ell og_{\sigma }\varepsilon_{2};$*

*(2) ${\left(\ell og_{\sigma }\varepsilon_{1}⊗\ell og_{\sigma }\varepsilon_{2}\right)}^{\beta }={\left(\ell og_{\sigma }\varepsilon_{1}\right)}^{\beta }⊗{\left(\ell og_{\sigma }\varepsilon_{2}\right)}^{\beta };$*

*(3) $\beta_{1}\ell og_{\sigma }\varepsilon_{1}⊕\beta_{2}\ell og_{\sigma }\varepsilon_{1}=\left(\beta_{1}+\beta_{2}\right)\ell og_{\sigma }\varepsilon_{1};$*

*(4) ${\left(\ell og_{\sigma }\varepsilon_{1}\right)}^{\beta_{1}}⊗{\left(\ell og_{\sigma }\varepsilon_{1}\right)}^{\beta_{2}}={\left(\ell og_{\sigma }\varepsilon_{1}\right)}^{\left(\beta_{1}+\beta_{2}\right)};$*

*(5) ${\left({\left(\ell og_{\sigma }\varepsilon_{1}\right)}^{\beta_{1}}\right)}^{\beta_{2}}={\left(\ell og_{\sigma }\varepsilon_{1}\right)}^{\beta_{1}\beta_{2}}.$*

**Proof.**  (1) Since, from Definition 23, we have

$$\ell og_{\sigma }\varepsilon_{1}⊕\ell og_{\sigma }\varepsilon_{2}=\left\{\begin{array}{c} \sqrt{1-{\left(\ell og_{\sigma }P_{\xi_{1}}\right)}^{2}\cdot {\left(\ell og_{\sigma }P_{\xi_{2}}\right)}^{2}},\\ \ell og_{\sigma }\left(\sqrt{1-I_{\xi_{1}}^{2}}\right)\cdot \ell og_{\sigma }\left(\sqrt{1-I_{\xi_{2}}^{2}}\right),\\ \ell og_{\sigma }\left(\sqrt{1-N_{\xi_{1}}^{2}}\right)\cdot \ell og_{\sigma }\left(\sqrt{1-N_{\xi_{2}}^{2}}\right) \end{array}\right\},$$

for any real number β>0, we obtain

$$\begin{array}{ccc} \beta \left(\ell og_{\sigma }\varepsilon_{1}⊕\ell og_{\sigma }\varepsilon_{2}\right) & = & \left\{\begin{array}{c} \sqrt{1-{\left({\left(\ell og_{\sigma }P_{\xi_{1}}\right)}^{2}\cdot {\left(\ell og_{\sigma }P_{\xi_{2}}\right)}^{2}\right)}^{\beta }},\\ {\left(\ell og_{\sigma }\left(\sqrt{1-I_{\xi_{1}}^{2}}\right)\cdot \ell og_{\sigma }\left(\sqrt{1-I_{\xi_{2}}^{2}}\right)\right)}^{\beta },\\ {\left(\ell og_{\sigma }\left(\sqrt{1-N_{\xi_{1}}^{2}}\right)\cdot \ell og_{\sigma }\left(\sqrt{1-N_{\xi_{2}}^{2}}\right)\right)}^{\beta } \end{array}\right\}\\ & = & \left\{\begin{array}{c} \sqrt{1-{\left({\left(\ell og_{\sigma }P_{\xi_{1}}\right)}^{2}\right)}^{\beta }},\\ {\left(\ell og_{\sigma }\left(\sqrt{1-I_{\xi_{1}}^{2}}\right)\right)}^{\beta },\\ {\left(\ell og_{\sigma }\left(\sqrt{1-N_{\xi_{1}}^{2}}\right)\right)}^{\beta } \end{array}\right\}⊕\left\{\begin{array}{c} \sqrt{1-{\left({\left(\ell og_{\sigma }P_{\xi_{2}}\right)}^{2}\right)}^{\beta }},\\ {\left(\ell og_{\sigma }\left(\sqrt{1-I_{\xi_{2}}^{2}}\right)\right)}^{\beta },\\ {\left(\ell og_{\sigma }\left(\sqrt{1-N_{\xi_{2}}^{2}}\right)\right)}^{\beta } \end{array}\right\}\\ & = & \beta \ell og_{\sigma }\varepsilon_{1}⊕\beta \ell og_{\sigma }\varepsilon_{2}. \end{array}$$

(2) Since, from Definition 23, we have

$$\begin{array}{cc} & \ell og_{\sigma }\varepsilon_{1}⊗\ell og_{\sigma }\varepsilon_{2}\\ = & \left\{\begin{array}{c} \sqrt{1-{\left(\ell og_{\sigma }P_{\xi_{1}}\right)}^{2}}\cdot \sqrt{1-{\left(\ell og_{\sigma }P_{\xi_{2}}\right)}^{2}},\\ \sqrt{1-{\left(1-\ell og_{\sigma }\left(\sqrt{1-I_{\xi_{1}}^{2}}\right)\right)}^{2}\cdot {\left(1-\ell og_{\sigma }\left(\sqrt{1-I_{\xi_{2}}^{2}}\right)\right)}^{2}},\\ \sqrt{1-{\left(1-\ell og_{\sigma }\left(\sqrt{1-N_{\xi_{1}}^{2}}\right)\right)}^{2}\cdot {\left(1-\ell og_{\sigma }\left(\sqrt{1-N_{\xi_{2}}^{2}}\right)\right)}^{2}} \end{array}\right\}, \end{array}$$

for any real number β>0, we obtain

$$\begin{array}{cc} & {\left(\ell og_{\sigma }\varepsilon_{1}⊗\ell og_{\sigma }\varepsilon_{2}\right)}^{\beta }\\ = & \left\{\begin{array}{c} {\left(\sqrt{1-{\left(\ell og_{\sigma }P_{\xi_{1}}\right)}^{2}}\right)}^{\beta }\cdot {\left(\sqrt{1-{\left(\ell og_{\sigma }P_{\xi_{2}}\right)}^{2}}\right)}^{\beta }\\ \sqrt{1-{\left(1-{\left(\ell og_{\sigma }\left(\sqrt{1-I_{\xi_{1}}^{2}}\right)\right)}^{2}\right)}^{\beta }\cdot {\left(1-{\left(\ell og_{\sigma }\left(\sqrt{1-I_{\xi_{2}}^{2}}\right)\right)}^{2}\right)}^{\beta }}\\ \sqrt{1-{\left(1-{\left(\ell og_{\sigma }\left(\sqrt{1-N_{\xi_{1}}^{2}}\right)\right)}^{2}\right)}^{\beta }\cdot {\left(1-{\left(\ell og_{\sigma }\left(\sqrt{1-N_{\xi_{2}}^{2}}\right)\right)}^{2}\right)}^{\beta }} \end{array}\right\}\\ = & {\left(\ell og_{\sigma }\varepsilon_{1}\right)}^{\beta }⊗{\left(\ell og_{\sigma }\varepsilon_{2}\right)}^{\beta }; \end{array}$$

(3) and (4) are similarly as above, so the procedure is eliminated here.

(5) Since, from Definition 23, we have

$$\begin{array}{ccc} {\left({\left(\ell og_{\sigma }\varepsilon_{1}\right)}^{\beta_{1}}\right)}^{\beta_{2}} & = & {\left(\begin{array}{c} {\left(\sqrt{1-{\left(\ell og_{\sigma }P_{\xi_{1}}\right)}^{2}}\right)}^{\beta_{1}}\\ \sqrt{1-{\left(1-{\left(\ell og_{\sigma }\left(\sqrt{1-I_{\xi_{1}}^{2}}\right)\right)}^{2}\right)}^{\beta_{1}}}\\ \sqrt{1-{\left(1-{\left(\ell og_{\sigma }\left(\sqrt{1-N_{\xi_{1}}^{2}}\right)\right)}^{2}\right)}^{\beta_{1}}} \end{array}\right)}^{\beta_{2}}\\ & = & \left(\begin{array}{c} {\left(\sqrt{1-{\left(\ell og_{\sigma }P_{\xi_{1}}\right)}^{2}}\right)}^{\beta_{1}\beta_{2}}\\ \sqrt{1-{\left(1-{\left(\ell og_{\sigma }\left(\sqrt{1-I_{\xi_{1}}^{2}}\right)\right)}^{2}\right)}^{\beta_{1}\beta_{2}}}\\ \sqrt{1-{\left(1-{\left(\ell og_{\sigma }\left(\sqrt{1-N_{\xi_{1}}^{2}}\right)\right)}^{2}\right)}^{\beta_{1}\beta_{2}}} \end{array}\right)\\ & = & {\left(\ell og_{\sigma }\varepsilon_{1}\right)}^{\beta_{1}\beta_{2}}, \end{array}$$

this is therefore proved. □

**Definition** **24.** *For any L-SFN, $\ell og_{\sigma }\varepsilon_{\rho }=\left\{\begin{array}{c} \sqrt{1-{\left(\ell og_{\sigma }P_{\xi_{\rho }}\left({\overset{ˇ}{r}}_{\gamma }\right)\right)}^{2}},\\ \ell og_{\sigma }\left(\sqrt{1-I_{\xi_{\rho }}^{2}\left({\overset{ˇ}{r}}_{\gamma }\right)}\right),\\ \ell og_{\sigma }\left(\sqrt{1-N_{\xi_{\rho }}^{2}\left({\overset{ˇ}{r}}_{\gamma }\right)}\right) \end{array}\right\}$ in ℜ. Then, score and accuracy values are defined as*

*(1) $\tilde{S}\left(\ell og_{\sigma }\varepsilon_{\rho }\right)=\left(1-{\left(\ell og_{\sigma }P_{\xi_{\rho }}\left({\overset{ˇ}{r}}_{\gamma }\right)\right)}^{2}\right)-{\left(\ell og_{\sigma }\left(\sqrt{1-I_{\xi_{\rho }}^{2}\left({\overset{ˇ}{r}}_{\gamma }\right)}\right)\right)}^{2}-{\left(\ell og_{\sigma }\left(\sqrt{1-N_{\xi_{\rho }}^{2}\left({\overset{ˇ}{r}}_{\gamma }\right)}\right)\right)}^{2},$*

*(2) $\tilde{A}\left(\ell og_{\sigma }\varepsilon_{\rho }\right)=\left(1-{\left(\ell og_{\sigma }P_{\xi_{\rho }}\left({\overset{ˇ}{r}}_{\gamma }\right)\right)}^{2}\right)+{\left(\ell og_{\sigma }\left(\sqrt{1-N_{\xi_{\rho }}^{2}\left({\overset{ˇ}{r}}_{\gamma }\right)}\right)\right)}^{2}.$*

The score and accuracy values defined above suggest which L-SFN is greater than other L-SFNs. The comparison technique is defined in the next definition.

**Definition** **25.** *For any L-SFN, $\ell og_{\sigma }\varepsilon_{\rho }=\left\{\begin{array}{c} \sqrt{1-{\left(\ell og_{\sigma }P_{\xi_{\rho }}\left({\overset{ˇ}{r}}_{\gamma }\right)\right)}^{2}},\\ \ell og_{\sigma }\left(\sqrt{1-I_{\xi_{\rho }}^{2}\left({\overset{ˇ}{r}}_{\gamma }\right)}\right),\\ \ell og_{\sigma }\left(\sqrt{1-N_{\xi_{\rho }}^{2}\left({\overset{ˇ}{r}}_{\gamma }\right)}\right) \end{array}\right\}$$\left(\rho =1,2\right)$ in ℜ. Then, the comparison technique is proposed as*

*(1) If $\tilde{S}\left(\ell og_{\sigma }\varepsilon_{1}\right)<\tilde{S}\left(\ell og_{\sigma }\varepsilon_{2}\right),$ then $\ell og_{\sigma }\varepsilon_{1}<\ell og_{\sigma }\varepsilon_{2},$*

*(2) If $\tilde{S}\left(\ell og_{\sigma }\varepsilon_{1}\right)>\tilde{S}\left(\ell og_{\sigma }\varepsilon_{2}\right),$ then $\ell og_{\sigma }\varepsilon_{1}>\ell og_{\sigma }\varepsilon_{2},$*

*(3) If $\tilde{S}\left(\ell og_{\sigma }\varepsilon_{1}\right)=\tilde{S}\left(\ell og_{\sigma }\varepsilon_{2}\right),$ then*

*(a) $\tilde{A}\left(\ell og_{\sigma }\varepsilon_{1}\right)<\tilde{A}\left(\ell og_{\sigma }\varepsilon_{2}\right),$ then $\ell og_{\sigma }\varepsilon_{1}<\ell og_{\sigma }\varepsilon_{2},$*

*(b) $\tilde{A}\left(\ell og_{\sigma }\varepsilon_{1}\right)>\tilde{A}\left(\ell og_{\sigma }\varepsilon_{2}\right),$ then $\ell og_{\sigma }\varepsilon_{1}>\ell og_{\sigma }\varepsilon_{2},$*

*(c) $\tilde{A}\left(\ell og_{\sigma }\varepsilon_{1}\right)=\tilde{A}\left(\ell og_{\sigma }\varepsilon_{2}\right),$ then $\ell og_{\sigma }\varepsilon_{1}\approx \ell og_{\sigma }\varepsilon_{2}.$*

## 5. Logarithmic Aggregation Operators for L-SFNs

Now, we propose novel spherical fuzzy logarithmic aggregation operators for L-SFNs based on defined spherical fuzzy logarithmic operations laws as follows:

### 5.1. Logarithmic Averaging Operators

**Definition** **26.** *For any collection of SFNs, $\varepsilon_{\rho }=\langle P_{\xi_{\rho }}\left({\overset{ˇ}{r}}_{\gamma }\right),I_{\xi_{\rho }}\left({\overset{ˇ}{r}}_{\gamma }\right),N_{\xi_{\rho }}\left({\overset{ˇ}{r}}_{\gamma }\right)\rangle$$\left(\rho =1,2,\ldots ,n\right)$ in ℜ, with $0<\sigma_{\rho }\leq \min\left\{P_{\xi_{\rho }},\sqrt{1-I_{\xi_{\rho }}^{2}},\sqrt{1-N_{\xi_{\rho }}^{2}}\right\}<1,$σ≠1. The structure of logarithmic spherical weighted averaging (L-SFWA) operator is*

$$L-SFWA\left(\varepsilon_{1},\varepsilon_{2},\ldots ,\varepsilon_{n}\right)=\sum_{\rho =1}^{n}\beta_{\rho \ell og\sigma_{\rho }}\varepsilon_{\rho },$$

*where $\beta_{\rho }\left(\rho =1,2,\ldots ,n\right)$ are weight vectors with $\beta_{\rho }\geq 0$ and $\sum_{\rho =1}^{n}\beta_{\rho }=1.$*

**Theorem** **6.** *For any collection of SFNs, $\varepsilon_{\rho }=\langle P_{\xi_{\rho }}\left({\overset{ˇ}{r}}_{\gamma }\right),I_{\xi_{\rho }}\left({\overset{ˇ}{r}}_{\gamma }\right),N_{\xi_{\rho }}\left({\overset{ˇ}{r}}_{\gamma }\right)\rangle$$\left(\rho =1,2,\ldots ,n\right)$ in ℜ, with $0<\sigma_{\rho }\leq \min\left\{P_{\xi_{\rho }},\sqrt{1-I_{\xi_{\rho }}^{2}},\sqrt{1-N_{\xi_{\rho }}^{2}}\right\}<1,$σ≠1. Then, by using logarithmic operations and Definition 26, L−SFWA is defined as*

$$\begin{array}{cc} & L-SFWA\left(\varepsilon_{1},\varepsilon_{2},\ldots ,\varepsilon_{n}\right),\\ = & \left\{\begin{array}{cc} \left(\begin{array}{c} \sqrt{1-\prod_{\rho =1}^{n}{\left(\ell og_{\sigma_{\rho }}P_{\xi_{\rho }}\right)}^{2\beta_{\rho }}},\\ \prod_{\rho =1}^{n}{\left(\ell og_{\sigma_{\rho }}\left(\sqrt{1-I_{\xi_{\rho }}^{2}}\right)\right)}^{\beta_{\rho }},\\ \prod_{\rho =1}^{n}{\left(\ell og_{\sigma_{\rho }}\left(\sqrt{1-N_{\xi_{\rho }}^{2}}\right)\right)}^{\beta_{\rho }} \end{array}\right) & 0<\sigma_{\rho }\leq \min\left\{\begin{array}{c} P_{\xi_{\rho }},\\ \sqrt{1-I_{\xi_{\rho }}^{2}},\\ \sqrt{1-N_{\xi_{\rho }}^{2}} \end{array}\right\}<1,\\ \left(\begin{array}{c} \sqrt{1-\prod_{\rho =1}^{n}{\left(\ell og_{\frac{1}{\sigma_{\rho }}}P_{\xi_{\rho }}\right)}^{2\beta_{\rho }}},\\ \prod_{\rho =1}^{n}{\left(\ell og_{\frac{1}{\sigma_{\rho }}}\left(\sqrt{1-I_{\xi_{\rho }}^{2}}\right)\right)}^{\beta_{\rho }},\\ \prod_{\rho =1}^{n}{\left(\ell og_{\frac{1}{\sigma_{\rho }}}\left(\sqrt{1-N_{\xi_{\rho }}^{2}}\right)\right)}^{\beta_{\rho }} \end{array}\right) & \begin{array}{c} 0<\frac{1}{\sigma_{\rho }}\leq \min\left\{\begin{array}{c} P_{\xi_{\rho }},\\ \sqrt{1-I_{\xi_{\rho }}^{2}},\\ \sqrt{1-N_{\xi_{\rho }}^{2}} \end{array}\right\}<1,\;\\ \sigma \neq 1, \end{array} \end{array}\right. \end{array}$$

*where $\beta_{\rho }\left(\rho =1,2,\ldots ,n\right)$ are weight vectors with $\beta_{\rho }\geq 0$ and $\sum_{\rho =1}^{n}\beta_{\rho }=1.$*

**Proof.**  Using mathematical induction to prove Theorem 6, we therefore proceed as follows:

(a) For n=2, since

$$\beta_{1}\ell og_{\sigma_{1}}\varepsilon_{1}=\left(\begin{array}{c} \sqrt{1-{\left({\left(\ell og_{\sigma_{1}}P_{\xi_{1}}\right)}^{2}\right)}^{\beta_{1}}},\\ {\left(\ell og_{\sigma_{1}}\left(\sqrt{1-I_{\xi_{1}}^{2}}\right)\right)}^{\beta_{1}},\\ {\left(\ell og_{\sigma_{1}}\left(\sqrt{1-N_{\xi_{1}}^{2}}\right)\right)}^{\beta_{1}} \end{array}\right)$$

and

$$\beta_{2}\ell og_{\sigma_{2}}\varepsilon_{2}=\left(\begin{array}{c} \sqrt{1-{\left({\left(\ell og_{\sigma_{2}}P_{\xi_{2}}\right)}^{2}\right)}^{\beta_{2}}},\\ {\left(\ell og_{\sigma_{2}}\left(\sqrt{1-I_{\xi_{2}}^{2}}\right)\right)}^{\beta_{2}},\\ {\left(\ell og_{\sigma_{2}}\left(\sqrt{1-N_{\xi_{2}}^{2}}\right)\right)}^{\beta_{2}} \end{array}\right).$$

Then,

$$\begin{array}{cc} & L-SFWA\left(\varepsilon_{1},\varepsilon_{2}\right)=\beta_{1}\ell og_{\sigma_{1}}\varepsilon_{1}⊕\beta_{2}\ell og_{\sigma_{2}}\varepsilon_{2}\\ = & \left(\begin{array}{c} \sqrt{1-{\left({\left(\ell og_{\sigma_{1}}P_{\xi_{1}}\right)}^{2}\right)}^{\beta_{1}}},\\ {\left(\ell og_{\sigma_{1}}\left(\sqrt{1-I_{\xi_{1}}^{2}}\right)\right)}^{\beta_{1}},\\ {\left(\ell og_{\sigma_{1}}\left(\sqrt{1-N_{\xi_{1}}^{2}}\right)\right)}^{\beta_{1}} \end{array}\right)⊕\left(\begin{array}{c} \sqrt{1-{\left({\left(\ell og_{\sigma_{2}}P_{\xi_{2}}\right)}^{2}\right)}^{\beta_{2}}},\\ {\left(\ell og_{\sigma_{2}}\left(\sqrt{1-I_{\xi_{2}}^{2}}\right)\right)}^{\beta_{2}},\\ {\left(\ell og_{\sigma_{2}}\left(\sqrt{1-N_{\xi_{2}}^{2}}\right)\right)}^{\beta_{2}} \end{array}\right)\\ = & \left(\begin{array}{c} \sqrt{1-{\left({\left(\ell og_{\sigma_{1}}P_{\xi_{1}}\right)}^{2}\right)}^{\beta_{1}}\cdot {\left({\left(\ell og_{\sigma_{2}}P_{\xi_{2}}\right)}^{2}\right)}^{\beta_{2}}},\\ {\left(\ell og_{\sigma_{1}}\left(\sqrt{1-I_{\xi_{1}}^{2}}\right)\right)}^{\beta_{1}}\cdot {\left(\ell og_{\sigma_{2}}\left(\sqrt{1-I_{\xi_{2}}^{2}}\right)\right)}^{\beta_{2}},\\ {\left(\ell og_{\sigma_{1}}\left(\sqrt{1-N_{\xi_{1}}^{2}}\right)\right)}^{\beta_{1}}\cdot {\left(\ell og_{\sigma_{2}}\left(\sqrt{1-N_{\xi_{2}}^{2}}\right)\right)}^{\beta_{2}} \end{array}\right)\\ = & \left(\begin{array}{c} \sqrt{1-\prod_{\rho =1}^{2}{\left(\ell og_{\sigma_{\rho }}P_{\xi_{\rho }}\right)}^{2\beta_{\rho }}},\\ \prod_{\rho =1}^{n}{\left(\ell og_{\sigma_{\rho }}\left(\sqrt{1-I_{\xi_{\rho }}^{2}}\right)\right)}^{\beta_{\rho }},\\ \prod_{\rho =1}^{n}{\left(\ell og_{\sigma_{\rho }}\left(\sqrt{1-N_{\xi_{\rho }}^{2}}\right)\right)}^{\beta_{\rho }} \end{array}\right). \end{array}$$

(b) Now, Theorem 6 is true for n=k,

$$L-SFWA\left(\varepsilon_{1},\varepsilon_{2},\ldots ,\varepsilon_{k}\right)=\left(\begin{array}{c} \sqrt{1-\prod_{\rho =1}^{k}{\left(\ell og_{\sigma_{\rho }}P_{\xi_{\rho }}\right)}^{2\beta_{\rho }}},\\ \prod_{\rho =1}^{k}{\left(\ell og_{\sigma_{\rho }}\left(\sqrt{1-I_{\xi_{\rho }}^{2}}\right)\right)}^{\beta_{\rho }},\\ \prod_{\rho =1}^{k}{\left(\ell og_{\sigma_{\rho }}\left(\sqrt{1-N_{\xi_{\rho }}^{2}}\right)\right)}^{\beta_{\rho }} \end{array}\right).$$

(c) Now, we prove that Theorem 6 for n=k+1, which is

$$L-SFWA\left(\varepsilon_{1},\varepsilon_{2},\ldots ,\varepsilon_{k},\varepsilon_{k+1}\right)=\sum_{\rho =1}^{k}\beta_{\rho }\ell og_{\sigma_{\rho }}\varepsilon_{\rho }+\beta_{k+1}\ell og_{\sigma_{k+1}}\varepsilon_{k+1}$$

$$\begin{array}{cc} & L-SFWA\left(\varepsilon_{1},\varepsilon_{2},\ldots ,\varepsilon_{k},\varepsilon_{k+1}\right)\\ = & \left(\begin{array}{c} \sqrt{1-\prod_{\rho =1}^{k}{\left(\ell og_{\sigma_{\rho }}P_{\xi_{\rho }}\right)}^{2\beta_{\rho }}},\\ \prod_{\rho =1}^{k}{\left(\ell og_{\sigma_{\rho }}\left(\sqrt{1-I_{\xi_{\rho }}^{2}}\right)\right)}^{\beta_{\rho }},\\ \prod_{\rho =1}^{k}{\left(\ell og_{\sigma_{\rho }}\left(\sqrt{1-N_{\xi_{\rho }}^{2}}\right)\right)}^{\beta_{\rho }} \end{array}\right)⊕\left(\begin{array}{c} \sqrt{1-{\left(\ell og_{\sigma_{k+1}}P_{\xi_{k+1}}\right)}^{2\beta_{k+1}}},\\ {\left(\ell og_{\sigma_{k+1}}\left(\sqrt{1-I_{\xi_{k+1}}^{2}}\right)\right)}^{\beta_{k+1}},\\ {\left(\ell og_{\sigma_{k+1}}\left(\sqrt{1-N_{\xi_{k+1}}^{2}}\right)\right)}^{\beta_{k+1}} \end{array}\right)\\ = & \left(\begin{array}{c} \sqrt{1-\prod_{\rho =1}^{k+1}{\left(\ell og_{\sigma_{\rho }}P_{\xi_{\rho }}\right)}^{2\beta_{\rho }}},\\ \prod_{\rho =1}^{k+1}{\left(\ell og_{\sigma_{\rho }}\left(\sqrt{1-I_{\xi_{\rho }}^{2}}\right)\right)}^{\beta_{\rho }},\\ \prod_{\rho =1}^{k+1}{\left(\ell og_{\sigma_{\rho }}\left(\sqrt{1-N_{\xi_{\rho }}^{2}}\right)\right)}^{\beta_{\rho }} \end{array}\right). \end{array}$$

Thus, Theorem 6 is true for n=z+1. Hence, it is satisfied for all *n*. Therefore,

$$L-SFWA\left(\varepsilon_{1},\varepsilon_{2},\ldots ,\varepsilon_{n}\right)=\left(\begin{array}{c} \sqrt{1-\prod_{\rho =1}^{n}{\left(\ell og_{\sigma_{\rho }}P_{\xi_{\rho }}\right)}^{2\beta_{\rho }}},\\ \prod_{\rho =1}^{n}{\left(\ell og_{\sigma_{\rho }}\left(\sqrt{1-I_{\xi_{\rho }}^{2}}\right)\right)}^{\beta_{\rho }},\\ \prod_{\rho =1}^{n}{\left(\ell og_{\sigma_{\rho }}\left(\sqrt{1-N_{\xi_{\rho }}^{2}}\right)\right)}^{\beta_{\rho }} \end{array}\right).$$

In a similar way, if $0<\frac{1}{\sigma_{\rho }}\leq \min\left\{\rho_{\xi_{\rho }},\sqrt{1-I_{\xi_{\rho }}^{2}},\sqrt{1-N_{\xi_{\rho }}^{2}}\right\}<1,$ σ≠1, we can also obtain

$$L-SFWA\left(\varepsilon_{1},\varepsilon_{2},\ldots ,\varepsilon_{n}\right)=\left(\begin{array}{c} \sqrt{1-\prod_{\rho =1}^{n}{\left(\ell og_{\frac{1}{\sigma_{\rho }}}P_{\xi_{\rho }}\right)}^{2\beta_{\rho }}},\\ \prod_{\rho =1}^{n}{\left(\ell og_{\frac{1}{\sigma_{\rho }}}\left(\sqrt{1-I_{\xi_{\rho }}^{2}}\right)\right)}^{\beta_{\rho }},\\ \prod_{\rho =1}^{n}{\left(\ell og_{\frac{1}{\sigma_{\rho }}}\left(\sqrt{1-N_{\xi_{\rho }}^{2}}\right)\right)}^{\beta_{\rho }} \end{array}\right),$$

which completes the proof. □

**Remark** **1.** *If $\sigma_{1}=\sigma_{2}=\sigma_{3}=\ldots =\sigma_{n}=\sigma ,$ that is $0<\sigma \leq \min\left\{P_{\xi_{\rho }},\sqrt{1-I_{\xi_{\rho }}^{2}},\sqrt{1-N_{\xi_{\rho }}^{2}}\right\}<1,$σ≠1, then the L−SFWA operator is reduced as follows:*

$$L-SFWA\left(\varepsilon_{1},\varepsilon_{2},\ldots ,\varepsilon_{n}\right)=\left(\begin{array}{c} \sqrt{1-\prod_{\rho =1}^{n}{\left(\ell og_{\sigma }P_{\xi_{\rho }}\right)}^{2\beta_{\rho }}},\\ \prod_{\rho =1}^{n}{\left(\ell og_{\sigma }\left(\sqrt{1-I_{\xi_{\rho }}^{2}}\right)\right)}^{\beta_{\rho }},\\ \prod_{\rho =1}^{n}{\left(\ell og_{\sigma }\left(\sqrt{1-N_{\xi_{\rho }}^{2}}\right)\right)}^{\beta_{\rho }}. \end{array}\right.$$

**Properties:** The L−SFWA operator satisfies some properties that are listed below:

(1) Idempotency: For any collection of SFNs, $\varepsilon_{\rho }=\langle P_{\xi_{\rho }}\left({\overset{ˇ}{r}}_{\gamma }\right),I_{\xi_{\rho }}\left({\overset{ˇ}{r}}_{\gamma }\right),N_{\xi_{\rho }}\left({\overset{ˇ}{r}}_{\gamma }\right)\rangle$ $\left(\rho =1,2,\ldots ,n\right)$ in *ℜ*. Then, if the collection of SFNs $\varepsilon_{\rho }=\langle P_{\xi_{\rho }}\left({\overset{ˇ}{r}}_{\gamma }\right),I_{\xi_{\rho }}\left({\overset{ˇ}{r}}_{\gamma }\right),N_{\xi_{\rho }}\left({\overset{ˇ}{r}}_{\gamma }\right)\rangle$ $\left(\rho =1,2,\ldots ,n\right)$ is identical,

$$L-SFWA\left(\varepsilon_{1},\varepsilon_{2},\ldots ,\varepsilon_{n}\right)=\varepsilon .$$

(2) Boundedness: For any collection of SFNs, $\varepsilon_{\rho }=\langle P_{\xi_{\rho }}\left({\overset{ˇ}{r}}_{\gamma }\right),I_{\xi_{\rho }}\left({\overset{ˇ}{r}}_{\gamma }\right),N_{\xi_{\rho }}\left({\overset{ˇ}{r}}_{\gamma }\right)\rangle$ $\left(\rho =1,2,\ldots ,n\right)$ in *ℜ*. $\varepsilon_{\rho }^{-}=\langle \min_{\rho }\rho_{\xi_{\rho }},\max_{\rho }I_{\xi_{\rho }},\max_{\rho }N_{\xi_{\rho }}\rangle$ and $\varepsilon_{\rho }^{+}=\langle \max_{\rho }P_{\xi_{\rho }},\min_{\rho }I_{\xi_{\rho }},\min_{\rho }N_{\xi_{\rho }}\rangle$ $\left(\rho =1,2,\ldots ,n\right)$ in *ℜ*, therefore

$$\varepsilon_{\rho }^{-}\subseteq L-SFWA\left(\varepsilon_{1},\varepsilon_{2},\ldots ,\varepsilon_{n}\right)\subseteq \varepsilon_{\rho }^{+}.$$

(3) Monotonically: For any collection of SFNs, $\varepsilon_{\rho }=\langle P_{\xi_{\rho }}\left({\overset{ˇ}{r}}_{\gamma }\right),I_{\xi_{\rho }}\left({\overset{ˇ}{r}}_{\gamma }\right),N_{\xi_{\rho }}\left({\overset{ˇ}{r}}_{\gamma }\right)\rangle$ $\left(\rho =1,2,\ldots ,n\right)$ in *ℜ*.If $\varepsilon_{\rho }\subseteq$ $\varepsilon_{\rho }^{*}$ for $\left(\rho =1,2,\ldots ,n\right),$ then

$$L-SFWA\left(\varepsilon_{1},\varepsilon_{2},\ldots ,\varepsilon_{n}\right)\subseteq L-SFWA\left(\varepsilon_{1}^{*},\varepsilon_{2}^{*},\ldots ,\varepsilon_{n}^{*}\right).$$

**Definition** **27.** *For any collection of SFNs, $\varepsilon_{\rho }=\langle P_{\xi_{\rho }}\left({\overset{ˇ}{r}}_{\gamma }\right),I_{\xi_{\rho }}\left({\overset{ˇ}{r}}_{\gamma }\right),N_{\xi_{\rho }}\left({\overset{ˇ}{r}}_{\gamma }\right)\rangle$$\left(\rho =1,2,\ldots ,n\right)$ in ℜ, with $0<\sigma_{\rho }\leq \min\left\{P_{\xi_{\rho }},\sqrt{1-I_{\xi_{\rho }}^{2}},\sqrt{1-N_{\xi_{\rho }}^{2}}\right\}<1,$σ≠1. The structure of the logarithmic spherical ordered weighted averaging (L-SFOWA) operator is*

$$L-SFOWA\left(\varepsilon_{1},\varepsilon_{2},\ldots ,\varepsilon_{n}\right)=\sum_{\rho =1}^{n}\beta_{\rho }\ell og_{\sigma_{\rho }}\varepsilon_{\eta \left(\rho \right),}$$

*where $\beta_{\rho }\left(\rho =1,2,\ldots ,n\right)$ are weight vectors with $\beta_{\rho }\geq 0$ and $\sum_{\rho =1}^{n}\beta_{\rho }=1$ and the ρth biggest weighted value is $\varepsilon_{\eta (\rho )}$ consequently by total order $\varepsilon_{\eta (1)}\geq \varepsilon_{\eta (2)}\geq \ldots \geq \varepsilon_{\eta (n)}.$*

**Theorem** **7.** *For any collection of SFNs, $\varepsilon_{\rho }=\langle P_{\xi_{\rho }}\left({\overset{ˇ}{r}}_{\gamma }\right),I_{\xi_{\rho }}\left({\overset{ˇ}{r}}_{\gamma }\right),N_{\xi_{\rho }}\left({\overset{ˇ}{r}}_{\gamma }\right)\rangle$$\left(\rho =1,2,\ldots ,n\right)$ in ℜ, with $0<\sigma_{\rho }\leq \min\left\{P_{\xi_{\rho }},\sqrt{1-I_{\xi_{\rho }}^{2}},\sqrt{1-N_{\xi_{\rho }}^{2}}\right\}<1,$σ≠1. Then, by using logarithmic operations and Definition 27, L−SFOWA is defined as*

(1) $$\begin{array}{cc} & L-SFOWA\left(\varepsilon_{1},\varepsilon_{2},\ldots ,\varepsilon_{n}\right)\\ = & \left\{\begin{array}{cc} \left(\begin{array}{c} \sqrt{1-\prod_{\rho =1}^{n}{\left(\ell og_{\sigma_{\rho }}P_{\xi_{\eta \left(\rho \right)}}\right)}^{2\beta_{\rho }}},\\ \prod_{\rho =1}^{n}{\left(\ell og_{\sigma_{\rho }}\left(\sqrt{1-I_{\xi_{\eta \left(\rho \right)}}^{2}}\right)\right)}^{\beta_{\rho }},\\ \prod_{\rho =1}^{n}{\left(\ell og_{\sigma_{\rho }}\left(\sqrt{1-N_{\xi_{\eta \left(\rho \right)}}^{2}}\right)\right)}^{\beta_{\rho }} \end{array}\right) & 0<\sigma_{\rho }\leq \min\left\{\begin{array}{c} P_{\xi_{\rho }},\\ \sqrt{1-I_{\xi_{\rho }}^{2}},\\ \sqrt{1-N_{\xi_{\rho }}^{2}} \end{array}\right\}<1,\\ \left(\begin{array}{c} \sqrt{1-\prod_{\rho =1}^{n}{\left(\ell og_{\frac{1}{\sigma_{\rho }}}P_{\xi_{\eta \left(\rho \right)}}\right)}^{2\beta_{\rho }}},\\ \prod_{\rho =1}^{n}{\left(\ell og_{\frac{1}{\sigma_{\rho }}}\left(\sqrt{1-I_{\xi_{\eta \left(\rho \right)}}^{2}}\right)\right)}^{\beta_{\rho }},\\ \prod_{\rho =1}^{n}{\left(\ell og_{\frac{1}{\sigma_{\rho }}}\left(\sqrt{1-N_{\xi_{\eta \left(\rho \right)}}^{2}}\right)\right)}^{\beta_{\rho }} \end{array}\right) & \begin{array}{c} 0<\frac{1}{\sigma_{\rho }}\leq \min\left\{\begin{array}{c} P_{\xi_{\rho }},\\ \sqrt{1-I_{\xi_{\rho }}^{2}},\\ \sqrt{1-N_{\xi_{\rho }}^{2}} \end{array}\right\}<1,\;\\ \sigma \neq 1, \end{array} \end{array}\right. \end{array}$$

*where $\beta_{\rho }\left(\rho =1,2,\ldots ,n\right)$ are weight vectors with $\beta_{\rho }\geq 0$ and $\sum_{\rho =1}^{n}\beta_{\rho }=1$ and the ρth biggest weighted value is $\varepsilon_{\eta (\rho )}$ consequently by total order $\varepsilon_{\eta (1)}\geq \varepsilon_{\eta (2)}\geq \ldots \geq \varepsilon_{\eta (n)}.$*

**Proof.**  The proof is similar to Theorem 6. Thus, the procedure is eliminated here. □

**Remark** **2.** *If $\sigma_{1}=\sigma_{2}=\sigma_{3}=\ldots =\sigma_{n}=\sigma ,$ that is, $0<\sigma \leq \min\left\{\rho_{\xi_{\rho }},\sqrt{1-I_{\xi_{\rho }}^{2}},\sqrt{1-N_{\xi_{\rho }}^{2}}\right\}<1,$σ≠1, then the L−SFOWA operator is reduced as follows:*

$$L-SFOWA\left(\varepsilon_{1},\varepsilon_{2},\ldots ,\varepsilon_{n}\right)=\left(\begin{array}{c} \sqrt{1-\prod_{\rho =1}^{n}{\left(\ell og_{\sigma }P_{\xi_{\eta \left(\rho \right)}}\right)}^{2\beta_{\rho }}},\\ \prod_{\rho =1}^{n}{\left(\ell og_{\sigma }\left(\sqrt{1-I_{\xi_{\eta \left(\rho \right)}}^{2}}\right)\right)}^{\beta_{\rho }},\\ \prod_{\rho =1}^{n}{\left(\ell og_{\sigma }\left(\sqrt{1-N_{\xi_{\eta \left(\rho \right)}}^{2}}\right)\right)}^{\beta_{\rho }} \end{array}\right).$$

**Properties:** The L−SFOWA operator satisfies some properties that are listed below:

(1) Idempotency: For any collection of SFNs, $\varepsilon_{\rho }=\langle P_{\xi_{\rho }}\left({\overset{ˇ}{r}}_{\gamma }\right),I_{\xi_{\rho }}\left({\overset{ˇ}{r}}_{\gamma }\right),N_{\xi_{\rho }}\left({\overset{ˇ}{r}}_{\gamma }\right)\rangle$ $\left(\rho =1,2,\ldots ,n\right)$ in *ℜ*.Then, if a collection of SFNs $\varepsilon_{\rho }=\langle P_{\xi_{\rho }}\left({\overset{ˇ}{r}}_{\gamma }\right),I_{\xi_{\rho }}\left({\overset{ˇ}{r}}_{\gamma }\right),N_{\xi_{\rho }}\left({\overset{ˇ}{r}}_{\gamma }\right)\rangle$ $\left(\rho =1,2,\ldots ,n\right)$ is identical, that is,

$$L-SFOWA\left(\varepsilon_{1},\varepsilon_{2},\ldots ,\varepsilon_{n}\right)=\varepsilon .$$

(2) Boundedness: For any collection of SFNs, $\varepsilon_{\rho }=\langle P_{\xi_{\rho }}\left({\overset{ˇ}{r}}_{\gamma }\right),I_{\xi_{\rho }}\left({\overset{ˇ}{r}}_{\gamma }\right),N_{\xi_{\rho }}\left({\overset{ˇ}{r}}_{\gamma }\right)\rangle$ $\left(\rho =1,2,\ldots ,n\right)$ in *ℜ*. $\varepsilon_{\rho }^{-}=\langle \min_{\rho }P_{\xi_{\rho }},\max_{\rho }I_{\xi_{\rho }},\max_{\rho }N_{\xi_{\rho }}\rangle$ and $\varepsilon_{\rho }^{+}=\langle \max_{\rho }P_{\xi_{\rho }},\min_{\rho }I_{\xi_{\rho }},\min_{\rho }N_{\xi_{\rho }}\rangle$ $\left(\rho =1,2,\ldots ,n\right)$ in *ℜ*; therefore,

$$\varepsilon_{\rho }^{-}\subseteq L-SFOWA\left(\varepsilon_{1},\varepsilon_{2},\ldots ,\varepsilon_{n}\right)\subseteq \varepsilon_{\rho }^{+}.$$

(3) Monotonically: For any collection of SFNs, $\varepsilon_{\rho }=\langle P_{\xi_{\rho }}\left({\overset{ˇ}{r}}_{\gamma }\right),I_{\xi_{\rho }}\left({\overset{ˇ}{r}}_{\gamma }\right),N_{\xi_{\rho }}\left({\overset{ˇ}{r}}_{\gamma }\right)\rangle$ $\left(\rho =1,2,\ldots ,n\right)$ in *ℜ*. If $\varepsilon_{\rho }\subseteq$ $\varepsilon_{\rho }^{*}$ for $\left(\rho =1,2,\ldots ,n\right),$ then

$$L-SFOWA\left(\varepsilon_{1},\varepsilon_{2},\ldots ,\varepsilon_{n}\right)\subseteq L-SFOWA\left(\varepsilon_{1}^{*},\varepsilon_{2}^{*},\ldots ,\varepsilon_{n}^{*}\right).$$

**Definition** **28.** *For any collection of SFNs, $\varepsilon_{\rho }=\langle P_{\xi_{\rho }}\left({\overset{ˇ}{r}}_{\gamma }\right),I_{\xi_{\rho }}\left({\overset{ˇ}{r}}_{\gamma }\right),N_{\xi_{\rho }}\left({\overset{ˇ}{r}}_{\gamma }\right)\rangle$$\left(\rho =1,2,\ldots ,n\right)$ in ℜ, with $0<\sigma_{\rho }\leq \min\left\{P_{\xi_{\rho }},\sqrt{1-I_{\xi_{\rho }}^{2}},\sqrt{1-N_{\xi_{\rho }}^{2}}\right\}<1,$σ≠1. The structure of a logarithmic spherical hybrid weighted averaging (L-SFHWA) operator is*

$$L-SFHWA\left(\varepsilon_{1},\varepsilon_{2},\ldots ,\varepsilon_{n}\right)=\sum_{\rho =1}^{n}\beta_{\rho }\ell og_{\sigma_{\rho }}\varepsilon_{\eta \left(\rho \right)}^{*},$$

where $\beta_{\rho }\left(\rho =1,2,\ldots ,n\right)$ are weight vectors with $\beta_{\rho }\geq 0$ and $\sum_{\rho =1}^{n}\beta_{\rho }=1$ and the ρth biggest weighted value is $\varepsilon_{\eta (\rho )}^{*}\left(\varepsilon_{\eta (\rho )}^{*}=n\beta_{\rho }\varepsilon_{\eta (\rho )},\;\rho \in N\right)$ consequently by total order $\varepsilon_{\eta (1)}^{*}\geq \varepsilon_{\eta (2)}^{*}\geq \ldots \geq \varepsilon_{\eta (n)}^{*}.$ In addition, associated weights are $\omega =(\omega_{1},\omega_{2},\ldots ,\omega_{n})$ with $\omega_{\rho }\geq 0$, $\Sigma_{\rho =1}^{n}\omega_{\rho }=1.$

**Theorem** **8.** *For any collection of SFNs, $\varepsilon_{\rho }=\langle P_{\xi_{\rho }}\left({\overset{ˇ}{r}}_{\gamma }\right),I_{\xi_{\rho }}\left({\overset{ˇ}{r}}_{\gamma }\right),N_{\xi_{\rho }}\left({\overset{ˇ}{r}}_{\gamma }\right)\rangle$$\left(\rho =1,2,\ldots ,n\right)$ in ℜ, with $0<\sigma_{\rho }\leq \min\left\{P_{\xi_{\rho }},\sqrt{1-I_{\xi_{\rho }}^{2}},\sqrt{1-N_{\xi_{\rho }}^{2}}\right\}<1,$σ≠1. Then, by using logarithmic operations and Definition 28, L−SFHWA is defined as*

(2) $$\begin{array}{cc} & L-SFHWA\left(\varepsilon_{1},\varepsilon_{2},\ldots ,\varepsilon_{n}\right),\\ = & \left\{\begin{array}{cc} \left(\begin{array}{c} \sqrt{1-\prod_{\rho =1}^{n}{\left(\ell og_{\sigma_{\rho }}P_{\xi_{\eta \left(\rho \right)}}^{*}\right)}^{2\beta_{\rho }}},\\ \prod_{\rho =1}^{n}{\left(\ell og_{\sigma_{\rho }}\left(\sqrt{1-{\left(I_{\xi_{\eta \left(\rho \right)}}^{*}\right)}^{2}}\right)\right)}^{\beta_{\rho }},\\ \prod_{\rho =1}^{n}{\left(\ell og_{\sigma_{\rho }}\left(\sqrt{1-{\left(N_{\xi_{\eta \left(\rho \right)}}^{*}\right)}^{2}}\right)\right)}^{\beta_{\rho }} \end{array}\right) & 0<\sigma_{\rho }\leq \min\left\{\begin{array}{c} P_{\xi_{\rho }},\\ \sqrt{1-I_{\xi_{\rho }}^{2}},\\ \sqrt{1-N_{\xi_{\rho }}^{2}} \end{array}\right\}<1,\\ \left(\begin{array}{c} \sqrt{1-\prod_{\rho =1}^{n}{\left(\ell og_{\frac{1}{\sigma_{\rho }}}P_{\xi_{\eta \left(\rho \right)}}^{*}\right)}^{2\beta_{\rho }}},\\ \prod_{\rho =1}^{n}{\left(\ell og_{\frac{1}{\sigma_{\rho }}}\left(\sqrt{1-{\left(I_{\xi_{\eta \left(\rho \right)}}^{*}\right)}^{2}}\right)\right)}^{\beta_{\rho }},\\ \prod_{\rho =1}^{n}{\left(\ell og_{\frac{1}{\sigma_{\rho }}}\left(\sqrt{1-{\left(N_{\xi_{\eta \left(\rho \right)}}^{*}\right)}^{2}}\right)\right)}^{\beta_{\rho }} \end{array}\right) & \begin{array}{c} 0<\frac{1}{\sigma_{\rho }}\leq \min\left\{\begin{array}{c} P_{\xi_{\rho }},\\ \sqrt{1-I_{\xi_{\rho }}^{2}},\\ \sqrt{1-N_{\xi_{\rho }}^{2}} \end{array}\right\}<1,\;\\ \sigma \neq 1, \end{array} \end{array}\right. \end{array}$$

where $\beta_{\rho }\left(\rho =1,2,\ldots ,n\right)$ are weight vectors with $\beta_{\rho }\geq 0$ and $\sum_{\rho =1}^{n}\beta_{\rho }=1$ and the ρth biggest weighted value is $\varepsilon_{\eta (\rho )}^{*}\left(\varepsilon_{\eta (\rho )}^{*}=n\beta_{\rho }\varepsilon_{\eta (\rho )},\;\rho \in N\right)$ consequently by total order $\varepsilon_{\eta (1)}^{*}\geq \varepsilon_{\eta (2)}^{*}\geq \ldots \geq \varepsilon_{\eta (n)}^{*}.$ In addition, associated weights are $\omega =(\omega_{1},\omega_{2},\ldots ,\omega_{n})$ with $\omega_{\rho }\geq 0$, $\Sigma_{\rho =1}^{n}\omega_{\rho }=1.$

**Proof.**  This proof issimilar to Theorem 6, so the procedure is eliminated here. □

**Remark** **3.** *If $\sigma_{1}=\sigma_{2}=\sigma_{3}=\ldots =\sigma_{n}=\sigma ,$ that is, $0<\sigma \leq \min\left\{P_{\xi_{\rho }},\sqrt{1-I_{\xi_{\rho }}^{2}},\sqrt{1-N_{\xi_{\rho }}^{2}}\right\}<1,$σ≠1, then the L−SFHWA operator reduces to*

$$L-SFHWA\left(\varepsilon_{1},\varepsilon_{2},\ldots ,\varepsilon_{n}\right)=\left(\begin{array}{c} \sqrt{1-\prod_{\rho =1}^{n}{\left(\ell og_{\sigma }P_{\xi_{\eta \left(\rho \right)}}^{*}\right)}^{2\beta_{\rho }}},\\ \prod_{\rho =1}^{n}{\left(\ell og_{\sigma }\left(\sqrt{1-{\left(I_{\xi_{\eta \left(\rho \right)}}^{*}\right)}^{2}}\right)\right)}^{\beta_{\rho }},\\ \prod_{\rho =1}^{n}{\left(\ell og_{\sigma }\left(\sqrt{1-{\left(N_{\xi_{\eta \left(\rho \right)}}^{*}\right)}^{2}}\right)\right)}^{\beta_{\rho }} \end{array}\right).$$

**Properties:** The L−SFHWA operator satisfies some properties that are listed below:

(1) Idempotency: For any collection of SFNs, $\varepsilon_{\rho }=\langle P_{\xi_{\rho }}\left({\overset{ˇ}{r}}_{\gamma }\right),I_{\xi_{\rho }}\left({\overset{ˇ}{r}}_{\gamma }\right),N_{\xi_{\rho }}\left({\overset{ˇ}{r}}_{\gamma }\right)\rangle$ $\left(\rho =1,2,\ldots ,n\right)$ in *ℜ*. Then, if a collection of SFNs $\varepsilon_{\rho }=\langle P_{\xi_{\rho }}\left({\overset{ˇ}{r}}_{\gamma }\right),I_{\xi_{\rho }}\left({\overset{ˇ}{r}}_{\gamma }\right),N_{\xi_{\rho }}\left({\overset{ˇ}{r}}_{\gamma }\right)\rangle$ $\left(\rho =1,2,\ldots ,n\right)$ are identical, that is,

$$L-SFHWA\left(\varepsilon_{1},\varepsilon_{2},\ldots ,\varepsilon_{n}\right)=\varepsilon .$$

(2) Boundedness: For any collection of SFNs, $\varepsilon_{\rho }=\langle P_{\xi_{\rho }}\left({\overset{ˇ}{r}}_{\gamma }\right),I_{\xi_{\rho }}\left({\overset{ˇ}{r}}_{\gamma }\right),N_{\xi_{\rho }}\left({\overset{ˇ}{r}}_{\gamma }\right)\rangle$ $\left(\rho =1,2,\ldots ,n\right)$ in *ℜ*. $\varepsilon_{\rho }^{-}=\langle \min_{\rho }P_{\xi_{\rho }},\max_{\rho }I_{\xi_{\rho }},\max_{\rho }N_{\xi_{\rho }}\rangle$ and $\varepsilon_{\rho }^{+}=\langle \max_{\rho }P_{\xi_{\rho }},\min_{\rho }I_{\xi_{\rho }},\min_{\rho }N_{\xi_{\rho }}\rangle$ $\left(\rho =1,2,\ldots ,n\right)$ in *ℜ*; therefore,

$$\varepsilon_{\rho }^{-}\subseteq L-SFHWA\left(\varepsilon_{1},\varepsilon_{2},\ldots ,\varepsilon_{n}\right)\subseteq \varepsilon_{\rho }^{+}.$$

(3) Monotonically: For any collection of SFNs, $\varepsilon_{\rho }=\langle P_{\xi_{\rho }}\left({\overset{ˇ}{r}}_{\gamma }\right),I_{\xi_{\rho }}\left({\overset{ˇ}{r}}_{\gamma }\right),N_{\xi_{\rho }}\left({\overset{ˇ}{r}}_{\gamma }\right)\rangle$ $\left(\rho =1,2,\ldots ,n\right)$ in *ℜ*.If $\varepsilon_{\rho }\subseteq$ $\varepsilon_{\rho }^{*}$ for $\left(\rho =1,2,\ldots ,n\right),$ then

$$L-SFHWA\left(\varepsilon_{1},\varepsilon_{2},\ldots ,\varepsilon_{n}\right)\subseteq L-SFHWA\left(\varepsilon_{1}^{*},\varepsilon_{2}^{*},\ldots ,\varepsilon_{n}^{*}\right).$$

### 5.2. Logarithmic Geometric Operators

**Definition** **29.** *For any collection of SFNs, $\varepsilon_{\rho }=\langle P_{\xi_{\rho }}\left({\overset{ˇ}{r}}_{\gamma }\right),I_{\xi_{\rho }}\left({\overset{ˇ}{r}}_{\gamma }\right),N_{\xi_{\rho }}\left({\overset{ˇ}{r}}_{\gamma }\right)\rangle$$\left(\rho =1,2,\ldots ,n\right)$ in ℜ, with $0<\sigma_{\rho }\leq \min\left\{P_{\xi_{\rho }},\sqrt{1-I_{\xi_{\rho }}^{2}},\sqrt{1-N_{\xi_{\rho }}^{2}}\right\}<1,$σ≠1. The structure of logarithmic spherical weighted geometric (L-SFWG) operator is*

$$L-SFWG\left(\varepsilon_{1},\varepsilon_{2},\ldots ,\varepsilon_{n}\right)=\prod_{\rho =1}^{n}{\left(\ell og_{\sigma_{\rho }}\varepsilon_{\rho }\right)}^{\beta_{\rho }},$$

*where $\beta_{\rho }\left(\rho =1,2,\ldots ,n\right)$ are weight vectors with $\beta_{\rho }\geq 0$ and $\sum_{\rho =1}^{n}\beta_{\rho }=1.$*

**Theorem** **9.** *For any collection of SFNs, $\varepsilon_{\rho }=\langle P_{\xi_{\rho }}\left({\overset{ˇ}{r}}_{\gamma }\right),I_{\xi_{\rho }}\left({\overset{ˇ}{r}}_{\gamma }\right),N_{\xi_{\rho }}\left({\overset{ˇ}{r}}_{\gamma }\right)\rangle$$\left(\rho =1,2,\ldots ,n\right)$ in ℜ, with $0<\sigma_{\rho }\leq \min\left\{P_{\xi_{\rho }},\sqrt{1-I_{\xi_{\rho }}^{2}},\sqrt{1-N_{\xi_{\rho }}^{2}}\right\}<1,$σ≠1. Then, by using logarithmic operations and Definition 29, L−SFWG is defined as*

$$\begin{array}{cc} & L-SFWG\left(\varepsilon_{1},\varepsilon_{2},\ldots ,\varepsilon_{n}\right)\\ = & \left\{\begin{array}{cc} \left(\begin{array}{c} \prod_{\rho =1}^{n}{\left(\sqrt{1-{\left(\ell og_{\sigma_{\rho }}P_{\xi_{\rho }}\right)}^{2}}\right)}^{\beta_{\rho }}\\ \sqrt{1-\prod_{\rho =1}^{n}{\left(1-{\left(\ell og_{\sigma_{\rho }}\left(\sqrt{1-I_{\xi_{\rho }}^{2}}\right)\right)}^{2}\right)}^{\beta_{\rho }}}\\ \sqrt{1-\prod_{\rho =1}^{n}{\left(1-{\left(\ell og_{\sigma_{\rho }}\left(\sqrt{1-N_{\xi_{\rho }}^{2}}\right)\right)}^{2}\right)}^{\beta_{\rho }}} \end{array}\right) & 0<\sigma_{\rho }\leq \min\left\{\begin{array}{c} P_{\xi_{\rho }},\\ \sqrt{1-I_{\xi_{\rho }}^{2}},\\ \sqrt{1-N_{\xi_{\rho }}^{2}} \end{array}\right\}<1,\\ \left(\begin{array}{c} \prod_{\rho =1}^{n}{\left(\sqrt{1-{\left(\ell og_{\frac{1}{\sigma_{\rho }}}P_{\xi_{\rho }}\right)}^{2}}\right)}^{\beta_{\rho }}\\ \sqrt{1-\prod_{\rho =1}^{n}{\left(1-{\left(\ell og_{\frac{1}{\sigma_{\rho }}}\left(\sqrt{1-I_{\xi_{\rho }}^{2}}\right)\right)}^{2}\right)}^{\beta_{\rho }}}\\ \sqrt{1-\prod_{\rho =1}^{n}{\left(1-{\left(\ell og_{\frac{1}{\sigma_{\rho }}}\left(\sqrt{1-N_{\xi_{\rho }}^{2}}\right)\right)}^{2}\right)}^{\beta_{\rho }}} \end{array}\right) & \begin{array}{c} 0<\frac{1}{\sigma_{\rho }}\leq \min\left\{\begin{array}{c} P_{\xi_{\rho }},\\ \sqrt{1-I_{\xi_{\rho }}^{2}},\\ \sqrt{1-N_{\xi_{\rho }}^{2}} \end{array}\right\}<1,\;\\ \sigma \neq 1, \end{array} \end{array}\right. \end{array}$$

*where $\beta_{\rho }\left(\rho =1,2,\ldots ,n\right)$ are weight vectors with $\beta_{\rho }\geq 0$ and $\sum_{\rho =1}^{n}\beta_{\rho }=1.$*

**Proof.**  Using mathematical induction to prove Theorem 9, we proceed as follows:

(a) For n=2, since

$${\left(\ell og_{\sigma_{1}}\varepsilon_{1}\right)}^{\beta_{1}}=\left(\begin{array}{c} {\left(\sqrt{1-{\left(\ell og_{\sigma_{1}}P_{\xi_{1}}\right)}^{2}}\right)}^{\beta_{1}}\\ \sqrt{1-{\left(1-{\left(\ell og_{\sigma_{1}}\left(\sqrt{1-I_{\xi_{1}}^{2}}\right)\right)}^{2}\right)}^{\beta_{1}}}\\ \sqrt{1-{\left(1-{\left(\ell og_{\sigma_{1}}\left(\sqrt{1-N_{\xi_{1}}^{2}}\right)\right)}^{2}\right)}^{\beta_{1}}} \end{array}\right)$$

and

$${\left(\ell og_{\sigma_{2}}\varepsilon_{2}\right)}^{\beta_{2}}=\left(\begin{array}{c} {\left(\sqrt{1-{\left(\ell og_{\sigma_{2}}P_{\xi_{2}}\right)}^{2}}\right)}^{\beta_{2}}\\ \sqrt{1-{\left(1-{\left(\ell og_{\sigma_{2}}\left(\sqrt{1-I_{\xi_{2}}^{2}}\right)\right)}^{2}\right)}^{\beta_{2}}}\\ \sqrt{1-{\left(1-{\left(\ell og_{\sigma_{2}}\left(\sqrt{1-N_{\xi_{2}}^{2}}\right)\right)}^{2}\right)}^{\beta_{2}}} \end{array}\right),$$

then

$$\begin{array}{cc} & L-SFWG\left(\varepsilon_{1},\varepsilon_{2}\right)={\left(\ell og_{\sigma_{1}}\varepsilon_{1}\right)}^{\beta_{1}}⊗{\left(\ell og_{\sigma_{2}}\varepsilon_{2}\right)}^{\beta_{2}}\\ = & \left(\begin{array}{c} {\left(\sqrt{1-{\left(\ell og_{\sigma_{1}}P_{\xi_{1}}\right)}^{2}}\right)}^{\beta_{1}}\\ \sqrt{1-{\left(1-{\left(\ell og_{\sigma_{1}}\left(\sqrt{1-I_{\xi_{1}}^{2}}\right)\right)}^{2}\right)}^{\beta_{1}}}\\ \sqrt{1-{\left(1-{\left(\ell og_{\sigma_{1}}\left(\sqrt{1-N_{\xi_{1}}^{2}}\right)\right)}^{2}\right)}^{\beta_{1}}} \end{array}\right)⊕\\ & \left(\begin{array}{c} {\left(\sqrt{1-{\left(\ell og_{\sigma_{2}}P_{\xi_{2}}\right)}^{2}}\right)}^{\beta_{2}}\\ \sqrt{1-{\left(1-{\left(\ell og_{\sigma_{2}}\left(\sqrt{1-I_{\xi_{2}}^{2}}\right)\right)}^{2}\right)}^{\beta_{2}}}\\ \sqrt{1-{\left(1-{\left(\ell og_{\sigma_{2}}\left(\sqrt{1-N_{\xi_{2}}^{2}}\right)\right)}^{2}\right)}^{\beta_{2}}} \end{array}\right)\\ = & \left\{\begin{array}{c} {\left(\sqrt{1-{\left(\ell og_{\sigma_{1}}P_{\xi_{1}}\right)}^{2}}\right)}^{\beta_{1}}\cdot {\left(\sqrt{1-{\left(\ell og_{\sigma_{2}}P_{\xi_{2}}\right)}^{2}}\right)}^{\beta_{2}}\\ \sqrt{1-{\left(1-{\left(\ell og_{\sigma_{1}}\left(\sqrt{1-I_{\xi_{1}}^{2}}\right)\right)}^{2}\right)}^{\beta_{1}}\cdot {\left(1-{\left(\ell og_{\sigma_{2}}\left(\sqrt{1-I_{\xi_{2}}^{2}}\right)\right)}^{2}\right)}^{\beta_{2}}}\\ \sqrt{1-{\left(1-{\left(\ell og_{\sigma_{1}}\left(\sqrt{1-N_{\xi_{1}}^{2}}\right)\right)}^{2}\right)}^{\beta_{1}}\cdot {\left(1-{\left(\ell og_{\sigma_{2}}\left(\sqrt{1-N_{\xi_{2}}^{2}}\right)\right)}^{2}\right)}^{\beta_{2}}} \end{array}\right\}\\ = & \left(\begin{array}{c} \prod_{\rho =1}^{2}{\left(\sqrt{1-{\left(\ell og_{\sigma_{\rho }}P_{\xi_{\rho }}\right)}^{2}}\right)}^{\beta_{\rho }}\\ \sqrt{1-\prod_{\rho =1}^{2}{\left(1-{\left(\ell og_{\sigma_{\rho }}\left(\sqrt{1-I_{\xi_{\rho }}^{2}}\right)\right)}^{2}\right)}^{\beta_{\rho }}}\\ \sqrt{1-\prod_{\rho =1}^{2}{\left(1-{\left(\ell og_{\sigma_{\rho }}\left(\sqrt{1-N_{\xi_{\rho }}^{2}}\right)\right)}^{2}\right)}^{\beta_{\rho }}} \end{array}\right). \end{array}$$

(b) Now, Theorem 9 is true for n=k,

$$L-SFWG\left(\varepsilon_{1},\varepsilon_{2},\ldots ,\varepsilon_{k}\right)=\left(\begin{array}{c} \prod_{\rho =1}^{k}{\left(\sqrt{1-{\left(\ell og_{\sigma_{\rho }}P_{\xi_{\rho }}\right)}^{2}}\right)}^{\beta_{\rho }}\\ \sqrt{1-\prod_{\rho =1}^{k}{\left(1-{\left(\ell og_{\sigma_{\rho }}\left(\sqrt{1-I_{\xi_{\rho }}^{2}}\right)\right)}^{2}\right)}^{\beta_{\rho }}}\\ \sqrt{1-\prod_{\rho =1}^{k}{\left(1-{\left(\ell og_{\sigma_{\rho }}\left(\sqrt{1-N_{\xi_{\rho }}^{2}}\right)\right)}^{2}\right)}^{\beta_{\rho }}} \end{array}\right).$$

(c) Now, we prove that Theorem 9 for n=k+1, that is,

$$L-SFWG\left(\varepsilon_{1},\varepsilon_{2},\ldots ,\varepsilon_{k},\varepsilon_{k+1}\right)=\prod_{\rho =1}^{k}{\left(\ell og_{\sigma_{\rho }}\varepsilon_{\rho }\right)}^{\beta_{\rho }}⊗{\left(\ell og_{\sigma_{k+1}}\varepsilon_{k+1}\right)}^{\beta_{k+1}}$$

$$\begin{array}{cc} & L-SFWG\left(\varepsilon_{1},\varepsilon_{2},\ldots ,\varepsilon_{k},\varepsilon_{k+1}\right)\\ = & \left(\begin{array}{c} \prod_{\rho =1}^{k}{\left(\sqrt{1-{\left(\ell og_{\sigma_{\rho }}P_{\xi_{\rho }}\right)}^{2}}\right)}^{\beta_{\rho }}\\ \sqrt{1-\prod_{\rho =1}^{k}{\left(1-{\left(\ell og_{\sigma_{\rho }}\left(\sqrt{1-I_{\xi_{\rho }}^{2}}\right)\right)}^{2}\right)}^{\beta_{\rho }}}\\ \sqrt{1-\prod_{\rho =1}^{k}{\left(1-{\left(\ell og_{\sigma_{\rho }}\left(\sqrt{1-N_{\xi_{\rho }}^{2}}\right)\right)}^{2}\right)}^{\beta_{\rho }}} \end{array}\right)\\ & ⊗\left(\begin{array}{c} {\left(\sqrt{1-{\left(\ell og_{\sigma_{k+1}}P_{\xi_{k+1}}\right)}^{2}}\right)}^{\beta_{k+1}}\\ \sqrt{1-{\left(1-{\left(\ell og_{\sigma_{k+1}}\left(\sqrt{1-I_{\xi_{k+1}}^{2}}\right)\right)}^{2}\right)}^{\beta_{k+1}}}\\ \sqrt{1-{\left(1-{\left(\ell og_{\sigma_{k+1}}\left(\sqrt{1-N_{\xi_{k+1}}^{2}}\right)\right)}^{2}\right)}^{\beta_{k+1}}} \end{array}\right)\\ = & \left(\begin{array}{c} \prod_{\rho =1}^{k+1}{\left(\sqrt{1-{\left(\ell og_{\sigma_{\rho }}\rho_{\xi_{\rho }}\right)}^{2}}\right)}^{\beta_{\rho }}\\ \sqrt{1-\prod_{\rho =1}^{k+1}{\left(1-{\left(\ell og_{\sigma_{\rho }}\left(\sqrt{1-I_{\xi_{\rho }}^{2}}\right)\right)}^{2}\right)}^{\beta_{\rho }}}\\ \sqrt{1-\prod_{\rho =1}^{k+1}{\left(1-{\left(\ell og_{\sigma_{\rho }}\left(\sqrt{1-N_{\xi_{\rho }}^{2}}\right)\right)}^{2}\right)}^{\beta_{\rho }}} \end{array}\right). \end{array}$$

Thus, Theorem 9 is true for n=z+1. Hence, it is satisfied for all *n*. Therefore,

$$L-SFWG\left(\varepsilon_{1},\varepsilon_{2},\ldots ,\varepsilon_{n}\right)=\left(\begin{array}{c} \prod_{\rho =1}^{n}{\left(\sqrt{1-{\left(\ell og_{\sigma_{\rho }}P_{\xi_{\rho }}\right)}^{2}}\right)}^{\beta_{\rho }}\\ \sqrt{1-\prod_{\rho =1}^{n}{\left(1-{\left(\ell og_{\sigma_{\rho }}\left(\sqrt{1-I_{\xi_{\rho }}^{2}}\right)\right)}^{2}\right)}^{\beta_{\rho }}}\\ \sqrt{1-\prod_{\rho =1}^{n}{\left(1-{\left(\ell og_{\sigma_{\rho }}\left(\sqrt{1-N_{\xi_{\rho }}^{2}}\right)\right)}^{2}\right)}^{\beta_{\rho }}} \end{array}\right).$$

In a similar way, if $0<\frac{1}{\sigma_{\rho }}\leq \min\left\{\rho_{\xi_{\rho }},\sqrt{1-I_{\xi_{\rho }}^{2}},\sqrt{1-N_{\xi_{\rho }}^{2}}\right\}<1,$ σ≠1, we can also obtain

$$L-SFWG\left(\varepsilon_{1},\varepsilon_{2},\ldots ,\varepsilon_{n}\right)=\left(\begin{array}{c} \prod_{\rho =1}^{n}{\left(\sqrt{1-{\left(\ell og_{\frac{1}{\sigma_{\rho }}}P_{\xi_{\rho }}\right)}^{2}}\right)}^{\beta_{\rho }}\\ \sqrt{1-\prod_{\rho =1}^{n}{\left(1-{\left(\ell og_{\frac{1}{\sigma_{\rho }}}\left(\sqrt{1-I_{\xi_{\rho }}^{2}}\right)\right)}^{2}\right)}^{\beta_{\rho }}}\\ \sqrt{1-\prod_{\rho =1}^{n}{\left(1-{\left(\ell og_{\frac{1}{\sigma_{\rho }}}\left(\sqrt{1-N_{\xi_{\rho }}^{2}}\right)\right)}^{2}\right)}^{\beta_{\rho }}} \end{array}\right),$$

which completes the proof. □

**Remark** **4.** *If $\sigma_{1}=\sigma_{2}=\sigma_{3}=\ldots =\sigma_{n}=\sigma ,$ that is, $0<\sigma \leq \min\left\{P_{\xi_{\rho }},\sqrt{1-I_{\xi_{\rho }}^{2}},\sqrt{1-N_{\xi_{\rho }}^{2}}\right\}<1,$σ≠1, then the L−SFWG operator reduces to*

$$L-SFWG\left(\varepsilon_{1},\varepsilon_{2},\ldots ,\varepsilon_{n}\right)=\left(\begin{array}{c} \prod_{\rho =1}^{n}{\left(\sqrt{1-{\left(\ell og_{\sigma }P_{\xi_{\rho }}\right)}^{2}}\right)}^{\beta_{\rho }}\\ \sqrt{1-\prod_{\rho =1}^{n}{\left(1-{\left(\ell og_{\sigma }\left(\sqrt{1-I_{\xi_{\rho }}^{2}}\right)\right)}^{2}\right)}^{\beta_{\rho }}}\\ \sqrt{1-\prod_{\rho =1}^{n}{\left(1-{\left(\ell og_{\sigma }\left(\sqrt{1-N_{\xi_{\rho }}^{2}}\right)\right)}^{2}\right)}^{\beta_{\rho }}} \end{array}\right).$$

**Properties:** The L−SFWG operator satisfies some properties that are listed below:

(1) Idempotency: For any collection of SFNs, $\varepsilon_{\rho }=\langle P_{\xi_{\rho }}\left({\overset{ˇ}{r}}_{\gamma }\right),I_{\xi_{\rho }}\left({\overset{ˇ}{r}}_{\gamma }\right),N_{\xi_{\rho }}\left({\overset{ˇ}{r}}_{\gamma }\right)\rangle$ $\left(\rho =1,2,\ldots ,n\right)$ in *ℜ*.Then, if a collection of SFNs $\varepsilon_{\rho }=\langle P_{\xi_{\rho }}\left({\overset{ˇ}{r}}_{\gamma }\right),I_{\xi_{\rho }}\left({\overset{ˇ}{r}}_{\gamma }\right),N_{\xi_{\rho }}\left({\overset{ˇ}{r}}_{\gamma }\right)\rangle$ $\left(\rho =1,2,\ldots ,n\right)$ are identical, that is,

$$L-SFWG\left(\varepsilon_{1},\varepsilon_{2},\ldots ,\varepsilon_{n}\right)=\varepsilon .$$

(2) Boundedness: For any collection of SFNs, $\varepsilon_{\rho }=\langle P_{\xi_{\rho }}\left({\overset{ˇ}{r}}_{\gamma }\right),I_{\xi_{\rho }}\left({\overset{ˇ}{r}}_{\gamma }\right),N_{\xi_{\rho }}\left({\overset{ˇ}{r}}_{\gamma }\right)\rangle$ $\left(\rho =1,2,\ldots ,n\right)$ in *ℜ*. $\varepsilon_{\rho }^{-}=\langle \min_{\rho }\rho_{\xi_{\rho }},\max_{\rho }I_{\xi_{\rho }},\max_{\rho }N_{\xi_{\rho }}\rangle$ and $\varepsilon_{\rho }^{+}=\langle \max_{\rho }P_{\xi_{\rho }},\min_{\rho }I_{\xi_{\rho }},\min_{\rho }N_{\xi_{\rho }}\rangle$ $\left(\rho =1,2,\ldots ,n\right)$ in *ℜ*, therefore

$$\varepsilon_{\rho }^{-}\subseteq L-SFWG\left(\varepsilon_{1},\varepsilon_{2},\ldots ,\varepsilon_{n}\right)\subseteq \varepsilon_{\rho }^{+}.$$

(3) Monotonically: For any collection of SFNs, $\varepsilon_{\rho }=\langle P_{\xi_{\rho }}\left({\overset{ˇ}{r}}_{\gamma }\right),I_{\xi_{\rho }}\left({\overset{ˇ}{r}}_{\gamma }\right),N_{\xi_{\rho }}\left({\overset{ˇ}{r}}_{\gamma }\right)\rangle$ $\left(\rho =1,2,\ldots ,n\right)$ in *ℜ*.If $\varepsilon_{\rho }\subseteq$ $\varepsilon_{\rho }^{*}$ for $\left(\rho =1,2,\ldots ,n\right),$ then

$$L-SFWG\left(\varepsilon_{1},\varepsilon_{2},\ldots ,\varepsilon_{n}\right)\subseteq L-SFWG\left(\varepsilon_{1}^{*},\varepsilon_{2}^{*},\ldots ,\varepsilon_{n}^{*}\right).$$

**Definition** **30.** *For any collection of SFNs, $\varepsilon_{\rho }=\langle P_{\xi_{\rho }}\left({\overset{ˇ}{r}}_{\gamma }\right),I_{\xi_{\rho }}\left({\overset{ˇ}{r}}_{\gamma }\right),N_{\xi_{\rho }}\left({\overset{ˇ}{r}}_{\gamma }\right)\rangle$$\left(\rho =1,2,\ldots ,n\right)$ in ℜ, with $0<\sigma_{\rho }\leq \min\left\{P_{\xi_{\rho }},\sqrt{1-I_{\xi_{\rho }}^{2}},\sqrt{1-N_{\xi_{\rho }}^{2}}\right\}<1,$σ≠1. The structure of logarithmic spherical ordered weighted geometric (L-SFOWG) operator is*

$$L-SFOWG\left(\varepsilon_{1},\varepsilon_{2},\ldots ,\varepsilon_{n}\right)=\prod_{\rho =1}^{n}{\left(\ell og_{\sigma_{\rho }}\varepsilon_{\eta \left(\rho \right)}\right)}^{\beta_{\rho }},$$

*where $\beta_{\rho }\left(\rho =1,2,\ldots ,n\right)$ are weight vectors with $\beta_{\rho }\geq 0$ and $\sum_{\rho =1}^{n}\beta_{\rho }=1$ and the ρth biggest weighted value is $\varepsilon_{\eta (\rho )}$ consequently by total order $\varepsilon_{\eta (1)}\geq \varepsilon_{\eta (2)}\geq \ldots \geq \varepsilon_{\eta (n)}.$*

**Theorem** **10.** *For any collection of SFNs, $\varepsilon_{\rho }=\langle P_{\xi_{\rho }}\left({\overset{ˇ}{r}}_{\gamma }\right),I_{\xi_{\rho }}\left({\overset{ˇ}{r}}_{\gamma }\right),N_{\xi_{\rho }}\left({\overset{ˇ}{r}}_{\gamma }\right)\rangle$$\left(\rho =1,2,\ldots ,n\right)$ in ℜ, with $0<\sigma_{\rho }\leq \min\left\{P_{\xi_{\rho }},\sqrt{1-I_{\xi_{\rho }}^{2}},\sqrt{1-N_{\xi_{\rho }}^{2}}\right\}<1,$σ≠1. Then, by using logarithmic operations and Definition 30, L−SFOWG defined as*

$$\begin{array}{cc} & L-SFOWG\left(\varepsilon_{1},\varepsilon_{2},\ldots ,\varepsilon_{n}\right)\\ = & \left\{\begin{array}{cc} \left(\begin{array}{c} \prod_{\rho =1}^{n}{\left(\sqrt{1-{\left(\ell og_{\sigma_{\rho }}P_{\xi_{\eta \left(\rho \right)}}\right)}^{2}}\right)}^{\beta_{\rho }}\\ \sqrt{1-\prod_{\rho =1}^{n}{\left(1-{\left(\ell og_{\sigma_{\rho }}\left(\sqrt{1-I_{\xi_{\eta \left(\rho \right)}}^{2}}\right)\right)}^{2}\right)}^{\beta_{\rho }}}\\ \sqrt{1-\prod_{\rho =1}^{n}{\left(1-{\left(\ell og_{\sigma_{\rho }}\left(\sqrt{1-N_{\xi_{\eta \left(\rho \right)}}^{2}}\right)\right)}^{2}\right)}^{\beta_{\rho }}} \end{array}\right) & 0<\sigma_{\rho }\leq \min\left\{\begin{array}{c} P_{\xi_{\rho }},\\ \sqrt{1-I_{\xi_{\rho }}^{2}},\\ \sqrt{1-N_{\xi_{\rho }}^{2}} \end{array}\right\}<1,\\ \left(\begin{array}{c} \prod_{\rho =1}^{n}{\left(\sqrt{1-{\left(\ell og_{\frac{1}{\sigma_{\rho }}}P_{\xi_{\eta \left(\rho \right)}}\right)}^{2}}\right)}^{\beta_{\rho }}\\ \sqrt{1-\prod_{\rho =1}^{n}{\left(1-{\left(\ell og_{\frac{1}{\sigma_{\rho }}}\left(\sqrt{1-I_{\xi_{\eta \left(\rho \right)}}^{2}}\right)\right)}^{2}\right)}^{\beta_{\rho }}}\\ \sqrt{1-\prod_{\rho =1}^{n}{\left(1-{\left(\ell og_{\frac{1}{\sigma_{\rho }}}\left(\sqrt{1-N_{\xi_{\eta \left(\rho \right)}}^{2}}\right)\right)}^{2}\right)}^{\beta_{\rho }}} \end{array}\right) & \begin{array}{c} 0<\frac{1}{\sigma_{\rho }}\leq \min\left\{\begin{array}{c} P_{\xi_{\rho }},\\ \sqrt{1-I_{\xi_{\rho }}^{2}},\\ \sqrt{1-N_{\xi_{\rho }}^{2}} \end{array}\right\}<1,\;\\ \sigma \neq 1, \end{array} \end{array}\right. \end{array}$$

*where $\beta_{\rho }\left(\rho =1,2,\ldots ,n\right)$ are weight vectors with $\beta_{\rho }\geq 0$ and $\sum_{\rho =1}^{n}\beta_{\rho }=1$ and the ρth biggest weighted value is $\varepsilon_{\eta (\rho )}$ consequently by total order $\varepsilon_{\eta (1)}\geq \varepsilon_{\eta (2)}\geq \ldots \geq \varepsilon_{\eta (n)}.$*

**Proof.**  This proof is similar to Theorem 9, so the procedure is eliminated here.□

**Remark** **5.** *If $\sigma_{1}=\sigma_{2}=\sigma_{3}=\ldots =\sigma_{n}=\sigma ,$ that is, $0<\sigma \leq \min\left\{P_{\xi_{\rho }},\sqrt{1-I_{\xi_{\rho }}^{2}},\sqrt{1-N_{\xi_{\rho }}^{2}}\right\}<1,$σ≠1, then L−SFOWG operator reduces to*

$$L-SFOWG\left(\varepsilon_{1},\varepsilon_{2},\ldots ,\varepsilon_{n}\right)=\left(\begin{array}{c} \prod_{\rho =1}^{n}{\left(\sqrt{1-{\left(\ell og_{\sigma }P_{\xi_{\eta \left(\rho \right)}}\right)}^{2}}\right)}^{\beta_{\rho }}\\ \sqrt{1-\prod_{\rho =1}^{n}{\left(1-{\left(\ell og_{\sigma }\left(\sqrt{1-I_{\xi_{\eta \left(\rho \right)}}^{2}}\right)\right)}^{2}\right)}^{\beta_{\rho }}}\\ \sqrt{1-\prod_{\rho =1}^{n}{\left(1-{\left(\ell og_{\sigma }\left(\sqrt{1-N_{\xi_{\eta \left(\rho \right)}}^{2}}\right)\right)}^{2}\right)}^{\beta_{\rho }}} \end{array}\right).$$

**Properties:** The L−SFOWG operator satisfies some properties that are listed below:

(1) Idempotency: For any collection of SFNs, $\varepsilon_{\rho }=\langle P_{\xi_{\rho }}\left({\overset{ˇ}{r}}_{\gamma }\right),I_{\xi_{\rho }}\left({\overset{ˇ}{r}}_{\gamma }\right),N_{\xi_{\rho }}\left({\overset{ˇ}{r}}_{\gamma }\right)\rangle$ $\left(\rho =1,2,\ldots ,n\right)$ in *ℜ*.Then, if the collection of SFNs $\varepsilon_{\rho }=\langle P_{\xi_{\rho }}\left({\overset{ˇ}{r}}_{\gamma }\right),I_{\xi_{\rho }}\left({\overset{ˇ}{r}}_{\gamma }\right),N_{\xi_{\rho }}\left({\overset{ˇ}{r}}_{\gamma }\right)\rangle$ $\left(\rho =1,2,\ldots ,n\right)$ is identical, that is,

$$L-SFOWG\left(\varepsilon_{1},\varepsilon_{2},\ldots ,\varepsilon_{n}\right)=\varepsilon .$$

(2) Boundedness: For any collection of SFNs, $\varepsilon_{\rho }=\langle P_{\xi_{\rho }}\left({\overset{ˇ}{r}}_{\gamma }\right),I_{\xi_{\rho }}\left({\overset{ˇ}{r}}_{\gamma }\right),N_{\xi_{\rho }}\left({\overset{ˇ}{r}}_{\gamma }\right)\rangle$ $\left(\rho =1,2,\ldots ,n\right)$ in *ℜ*. $\varepsilon_{\rho }^{-}=\langle \min_{\rho }P_{\xi_{\rho }},\max_{\rho }I_{\xi_{\rho }},\max_{\rho }N_{\xi_{\rho }}\rangle$ and $\varepsilon_{\rho }^{+}=\langle \max_{\rho }P_{\xi_{\rho }},\min_{\rho }I_{\xi_{\rho }},\min_{\rho }N_{\xi_{\rho }}\rangle$ $\left(\rho =1,2,\ldots ,n\right)$ in *ℜ*, therefore

$$\varepsilon_{\rho }^{-}\subseteq L-SFOWG\left(\varepsilon_{1},\varepsilon_{2},\ldots ,\varepsilon_{n}\right)\subseteq \varepsilon_{\rho }^{+}.$$

(3) Monotonically: For any collection of SFNs, $\varepsilon_{\rho }=\langle P_{\xi_{\rho }}\left({\overset{ˇ}{r}}_{\gamma }\right),I_{\xi_{\rho }}\left({\overset{ˇ}{r}}_{\gamma }\right),N_{\xi_{\rho }}\left({\overset{ˇ}{r}}_{\gamma }\right)\rangle$ $\left(\rho =1,2,\ldots ,n\right)$ in *ℜ*. If $\varepsilon_{\rho }\subseteq$ $\varepsilon_{\rho }^{*}$ for $\left(\rho =1,2,\ldots ,n\right),$ then

$$L-SFOWG\left(\varepsilon_{1},\varepsilon_{2},\ldots ,\varepsilon_{n}\right)\subseteq L-SFOWG\left(\varepsilon_{1}^{*},\varepsilon_{2}^{*},\ldots ,\varepsilon_{n}^{*}\right).$$

**Definition** **31.** *For any collection of SFNs, $\varepsilon_{\rho }=\langle P_{\xi_{\rho }}\left({\overset{ˇ}{r}}_{\gamma }\right),I_{\xi_{\rho }}\left({\overset{ˇ}{r}}_{\gamma }\right),N_{\xi_{\rho }}\left({\overset{ˇ}{r}}_{\gamma }\right)\rangle$$\left(\rho =1,2,\ldots ,n\right)$ in ℜ, with $0<\sigma_{\rho }\leq \min\left\{P_{\xi_{\rho }},\sqrt{1-I_{\xi_{\rho }}^{2}},\sqrt{1-N_{\xi_{\rho }}^{2}}\right\}<1,$σ≠1. The structure of logarithmic spherical hybrid weighted geometric (L-SFHWG) operator is*

$$L-SFHWG\left(\varepsilon_{1},\varepsilon_{2},\ldots ,\varepsilon_{n}\right)=\prod_{\rho =1}^{n}{\left(\ell og_{\sigma_{\rho }}\varepsilon_{\eta \left(\rho \right)}^{*}\right)}^{\beta_{\rho }},$$

where $\beta_{\rho }\left(\rho =1,2,\ldots ,n\right)$ are weight vectors with $\beta_{\rho }\geq 0$ and $\sum_{\rho =1}^{n}\beta_{\rho }=1$ and the ρth biggest weighted value is $\varepsilon_{\eta (\rho )}^{*}\left(\varepsilon_{\eta (\rho )}^{*}=n\beta_{\rho }\varepsilon_{\eta (\rho )},\;\rho \in N\right)$ consequently by total order $\varepsilon_{\eta (1)}^{*}\geq \varepsilon_{\eta (2)}^{*}\geq \ldots \geq \varepsilon_{\eta (n)}^{*}.$ In addition, associated weights are $\omega =(\omega_{1},\omega_{2},\ldots ,\omega_{n})$ with $\omega_{\rho }\geq 0$, $\Sigma_{\rho =1}^{n}\omega_{\rho }=1.$

**Theorem** **11.** *For any collection of SFNs, $\varepsilon_{\rho }=\langle P_{\xi_{\rho }}\left({\overset{ˇ}{r}}_{\gamma }\right),I_{\xi_{\rho }}\left({\overset{ˇ}{r}}_{\gamma }\right),N_{\xi_{\rho }}\left({\overset{ˇ}{r}}_{\gamma }\right)\rangle$$\left(\rho =1,2,\ldots ,n\right)$ in ℜ, with $0<\sigma_{\rho }\leq \min\left\{\rho_{\xi_{\rho }},\sqrt{1-I_{\xi_{\rho }}^{2}},\sqrt{1-N_{\xi_{\rho }}^{2}}\right\}<1,$σ≠1. Then, by using logarithmic operations and Definition 31, L−SFHWG is defined as*

(3) $$\begin{array}{cc} & L-SFHWG\left(\varepsilon_{1},\varepsilon_{2},\ldots ,\varepsilon_{n}\right)\\ = & \left\{\begin{array}{cc} \left(\begin{array}{c} \prod_{\rho =1}^{n}{\left(\sqrt{1-{\left(\ell og_{\sigma_{\rho }}P_{\xi_{\eta \left(\rho \right)}}^{*}\right)}^{2}}\right)}^{\beta_{\rho }}\\ \sqrt{1-\prod_{\rho =1}^{n}{\left(1-{\left(\ell og_{\sigma_{\rho }}\left(\sqrt{1-{\left(I_{\xi_{\eta \left(\rho \right)}}^{*}\right)}^{2}}\right)\right)}^{2}\right)}^{\beta_{\rho }}}\\ \sqrt{1-\prod_{\rho =1}^{n}{\left(1-{\left(\ell og_{\sigma_{\rho }}\left(\sqrt{1-{\left(N_{\xi_{\eta \left(\rho \right)}}^{*}\right)}^{2}}\right)\right)}^{2}\right)}^{\beta_{\rho }}} \end{array}\right) & 0<\sigma_{\rho }\leq \min\left\{\begin{array}{c} P_{\xi_{\rho }},\\ \sqrt{1-I_{\xi_{\rho }}^{2}},\\ \sqrt{1-N_{\xi_{\rho }}^{2}} \end{array}\right\}<1,\\ \left(\begin{array}{c} \prod_{\rho =1}^{n}{\left(\sqrt{1-{\left(\ell og_{\frac{1}{\sigma_{\rho }}}P_{\xi_{\eta \left(\rho \right)}}^{*}\right)}^{2}}\right)}^{\beta_{\rho }}\\ \sqrt{1-\prod_{\rho =1}^{n}{\left(1-{\left(\ell og_{\frac{1}{\sigma_{\rho }}}\left(\sqrt{1-{\left(I_{\xi_{\eta \left(\rho \right)}}^{*}\right)}^{2}}\right)\right)}^{2}\right)}^{\beta_{\rho }}}\\ \sqrt{1-\prod_{\rho =1}^{n}{\left(1-{\left(\ell og_{\frac{1}{\sigma_{\rho }}}\left(\sqrt{1-{\left(N_{\xi_{\eta \left(\rho \right)}}^{*}\right)}^{2}}\right)\right)}^{2}\right)}^{\beta_{\rho }}} \end{array}\right) & \begin{array}{c} 0<\frac{1}{\sigma_{\rho }}\leq \min\left\{\begin{array}{c} P_{\xi_{\rho }},\\ \sqrt{1-I_{\xi_{\rho }}^{2}},\\ \sqrt{1-N_{\xi_{\rho }}^{2}} \end{array}\right\}<1,\;\\ \sigma \neq 1, \end{array} \end{array}\right. \end{array}$$

where $\beta_{\rho }\left(\rho =1,2,\ldots ,n\right)$ are weight vectors with $\beta_{\rho }\geq 0$ and $\sum_{\rho =1}^{n}\beta_{\rho }=1$ and the ρth biggest weighted value is $\varepsilon_{\eta (\rho )}^{*}\left(\varepsilon_{\eta (\rho )}^{*}=n\beta_{\rho }\varepsilon_{\eta (\rho )},\;\rho \in N\right)$ consequently by total order $\varepsilon_{\eta (1)}^{*}\geq \varepsilon_{\eta (2)}^{*}\geq \ldots \geq \varepsilon_{\eta (n)}^{*}.$ In addition, the associated weights are $\omega =(\omega_{1},\omega_{2},\ldots ,\omega_{n})$ with $\omega_{\rho }\geq 0$, $\Sigma_{\rho =1}^{n}\omega_{\rho }=1.$

**Proof.**  This proof is similar to Theorem 9. Thus, the procedure is eliminated here. □

**Remark** **6.** *If $\sigma_{1}=\sigma_{2}=\sigma_{3}=\ldots =\sigma_{n}=\sigma ,$ that is, $0<\sigma \leq \min\left\{P_{\xi_{\rho }},\sqrt{1-I_{\xi_{\rho }}^{2}},\sqrt{1-N_{\xi_{\rho }}^{2}}\right\}<1,$σ≠1; then, L−SFHWG operator reduces to*

$$L-SFHWG\left(\varepsilon_{1},\varepsilon_{2},\ldots ,\varepsilon_{n}\right)=\left(\begin{array}{c} \prod_{\rho =1}^{n}{\left(\sqrt{1-{\left(\ell og_{\sigma }P_{\xi_{\eta \left(\rho \right)}}^{*}\right)}^{2}}\right)}^{\beta_{\rho }}\\ \sqrt{1-\prod_{\rho =1}^{n}{\left(1-{\left(\ell og_{\sigma }\left(\sqrt{1-{\left(I_{\xi_{\eta \left(\rho \right)}}^{*}\right)}^{2}}\right)\right)}^{2}\right)}^{\beta_{\rho }}}\\ \sqrt{1-\prod_{\rho =1}^{n}{\left(1-{\left(\ell og_{\sigma }\left(\sqrt{1-{\left(N_{\xi_{\eta \left(\rho \right)}}^{*}\right)}^{2}}\right)\right)}^{2}\right)}^{\beta_{\rho }}} \end{array}\right).$$

**Properties:** The L−SFHWG operator satisfies some properties that are listed below:

(1) Idempotency: For any collection of SFNs, $\varepsilon_{\rho }=\langle P_{\xi_{\rho }}\left({\overset{ˇ}{r}}_{\gamma }\right),I_{\xi_{\rho }}\left({\overset{ˇ}{r}}_{\gamma }\right),N_{\xi_{\rho }}\left({\overset{ˇ}{r}}_{\gamma }\right)\rangle$ $\left(\rho =1,2,\ldots ,n\right)$ in *ℜ*. Then, if collection of SFNs $\varepsilon_{\rho }=\langle P_{\xi_{\rho }}\left({\overset{ˇ}{r}}_{\gamma }\right),I_{\xi_{\rho }}\left({\overset{ˇ}{r}}_{\gamma }\right),N_{\xi_{\rho }}\left({\overset{ˇ}{r}}_{\gamma }\right)\rangle$ $\left(\rho =1,2,\ldots ,n\right)$ are identical, that is,

$$L-SFHWG\left(\varepsilon_{1},\varepsilon_{2},\ldots ,\varepsilon_{n}\right)=\varepsilon .$$

(2) Boundedness: For any collection of SFNs, $\varepsilon_{\rho }=\langle P_{\xi_{\rho }}\left({\overset{ˇ}{r}}_{\gamma }\right),I_{\xi_{\rho }}\left({\overset{ˇ}{r}}_{\gamma }\right),N_{\xi_{\rho }}\left({\overset{ˇ}{r}}_{\gamma }\right)\rangle$ $\left(\rho =1,2,\ldots ,n\right)$ in *ℜ*. $\varepsilon_{\rho }^{-}=\langle \min_{\rho }P_{\xi_{\rho }},\max_{\rho }I_{\xi_{\rho }},\max_{\rho }N_{\xi_{\rho }}\rangle$ and $\varepsilon_{\rho }^{+}=\langle \max_{\rho }\rho_{\xi_{\rho }},\min_{\rho }I_{\xi_{\rho }},\min_{\rho }N_{\xi_{\rho }}\rangle$ $\left(\rho =1,2,\ldots ,n\right)$ in *ℜ*, therefore

$$\varepsilon_{\rho }^{-}\subseteq L-SFHWG\left(\varepsilon_{1},\varepsilon_{2},\ldots ,\varepsilon_{n}\right)\subseteq \varepsilon_{\rho }^{+}.$$

(3) Monotonically: For any collection of SFNs, $\varepsilon_{\rho }=\langle P_{\xi_{\rho }}\left({\overset{ˇ}{r}}_{\gamma }\right),I_{\xi_{\rho }}\left({\overset{ˇ}{r}}_{\gamma }\right),N_{\xi_{\rho }}\left({\overset{ˇ}{r}}_{\gamma }\right)\rangle$ $\left(\rho =1,2,\ldots ,n\right)$ in *ℜ*. If $\varepsilon_{\rho }\subseteq$ $\varepsilon_{\rho }^{*}$ for $\left(\rho =1,2,\ldots ,n\right),$ then

$$L-SFHWG\left(\varepsilon_{1},\varepsilon_{2},\ldots ,\varepsilon_{n}\right)\subseteq L-SFHWG\left(\varepsilon_{1}^{*},\varepsilon_{2}^{*},\ldots ,\varepsilon_{n}^{*}\right).$$

## 6. Proposed Technique for Solving Decision-Making Problems

In this section, we propose a new approach to decision-making based on the spherical fuzzy set. This approach will use data information provided by the decision problem only and does not need any additional information provided by decision makers, in order to avoid the effect of subjective information influencing the decision results. In the following, we will introduce a spherical fuzzy set decision-making matrix as indicated below.

**Step 1**: Let $H=(h_{1},h_{2},\ldots ,h_{m})$ be a distinct set of *m* probable alternatives and $Y=(y_{1},y_{2},\ldots ,y_{n})$ be a finite set of *n* criteria, where $h_{i}$ indicates the ith alternatives and $y_{j}$ indicates the jth criteria. Let $D=(d_{1},d_{2},\ldots ,d_{t})$ be a finite set of *t* experts, where $d_{k}$ indicates the *k*th expert. The expert $d_{k}$ supplies her appraisal of an alternative $h_{i}$ on an attribute $y_{j}$ as a SFNs (i=1,2,…,m;j=1,2,…,n). The experts’ information is represented by the spherical fuzzy set decision-making matrix $D^{s}={\left[E_{i\rho }^{\left(s\right)}\right]}_{m\times n}$. Assume that $\beta_{\rho }\left(\rho =1,2,\ldots ,m\right)$ is a weight vector of the attribute $y_{j}$ such that $0\leq \beta_{\rho }\leq 1,$ $\sum_{\rho =1}^{n}\beta_{\rho }=1$ and $\psi =(\psi_{1},\psi_{2},\ldots ,\psi_{m})$ is the weight vector of the decision makers $d_{k}$ such that $\psi_{k}\leq 1,$ $\sum_{k=1}^{n}\psi_{k}=1.$
  When we construct the spherical fuzzy decision-making matrices, $D^{s}={\left[E_{i\rho }^{\left(s\right)}\right]}_{m\times n}$ for decisions. Basically, criteria have two types: one is benefit criteria and the other one is cost criteria. If the spherical fuzzy decision matrices have cost type criteria matrices, $D^{s}={\left[E_{i\rho }^{s}\right]}_{{}_{m\times n}}$ can be converted into the normalized spherical fuzzy decision matrices, ${\overset{ˇ}{r}}^{s}={\left[{\overset{ˇ}{r}}_{i\rho }^{\left(s\right)}\right]}_{{}_{m\times n}}$, where ${\overset{ˇ}{r}}_{ip}^{s}=\left\{\begin{array}{c} E_{i\rho }^{s},\;\mathrm{for}\;\mathrm{benefit}\;\mathrm{criteria}\;A_{p}\\ {\bar{E}}_{i\rho }^{s},\;\mathrm{for}\;\cos t\;\mathrm{criteria}\;A_{p}, \end{array}\right.j=1,2,\ldots ,n,$ and ${\bar{E}}_{i\rho }^{s}$ is the complement of $E_{i\rho }^{s}.$ If all the criteria have the same type, then there is no need for normalization.
  Taking the decision information from the given matrix ${\overset{ˇ}{r}}_{k}$ and using the SFWA/SFWG operator, the individual total spherical fuzzy preference value ${\overset{ˇ}{r}}_{i}^{k}$ of the alternative $h_{i}$ is derived as follows:
  $${\overset{ˇ}{r}}_{i}^{k}=L-SFWA\left(\varepsilon_{i1}^{k},\varepsilon_{i2}^{k},\ldots ,\varepsilon_{in}^{k}\right),\left(i=1,2,\ldots ,m;k=1,2,\ldots ,t\right),$$
  where $\beta ={\left(\beta_{1},\beta_{2},\ldots ,\beta_{n}\right)}^{T}$ is the weight vector of the attribute.
**Step 2**: In this step, we find the collective spherical information using a spherical fuzzy weighted averaging aggregation operator.
**Step 3**: In this step, we find the weights of each of the criteria by using the spherical fuzzy entropy:
  $$\gamma_{q}=\frac{1+\frac{1}{n}\sum_{\rho =1}^{n}\left(P_{i}\log\left(P_{i}\right)+I_{i}\log\left(I_{i}\right)+N_{i}\log\left(N_{i}\right)\right)}{\sum_{q=1}^{n}\left(1+\frac{1}{n}\sum_{\rho =1}^{n}P_{i}\log\left(P_{i}\right)+I_{i}\log\left(I_{i}\right)+N_{i}\log\left(N_{i}\right)\right)}.$$
**Step 4**: In this step, we calculate the aggregated information using all the logarithmic aggregation operators of spherical fuzzy sets.
**Step 5**: We find the score value $\tilde{S}\left(\ell og_{\sigma }\varepsilon_{\rho }\right)$ and the accuracy value $\tilde{A}\left(\ell og_{\sigma }\varepsilon_{\rho }\right)$ of the cumulative overall preference value $h_{i}$ (i=1,2,…,m).
**Step 6**: By the definition, rank the alternatives $h_{i}$ (i=1,2,…,m) and choose the best alternative that has the maximum score value.

**Figure 4:**
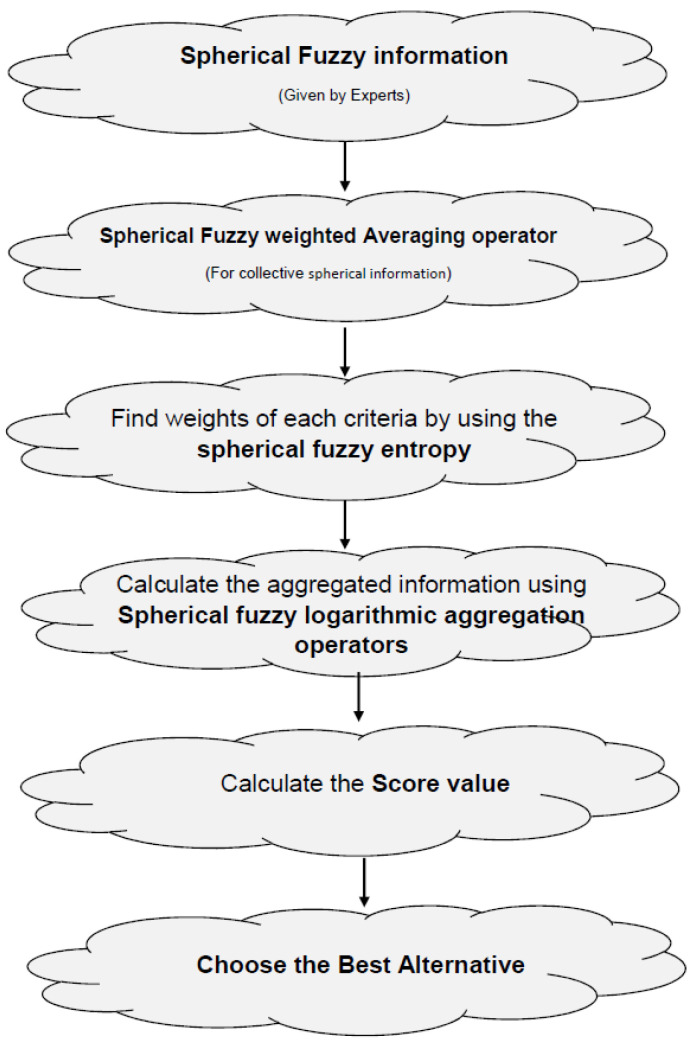
Algorithmic Steps.

The Algorithmic Steps are shown in Figure 4.

### 6.1. Numerical Example

Assume that there is a committee that selects five applicable emerging technology enterprises $H_{g}\left(g=1,2,3,4,5\right),$ which are given as follows:

(1) Augmented Reality $\left(H_{1}\right)$,

(2) Personalized Medicine $\left(H_{2}\right),$

(3) Artificial Intelligence $\left(H_{3}\right)$,

(4) Gene Drive $\left(H_{4}\right)$,

(5) Quantum Computing $\left(H_{5}\right)$

To assess the possible rising technology enterprises according to the four attributes, which are

(1) Advancement $\left(D_{1}\right)$,

(2) market risk $\left(D_{2}\right)$,

(3) financial investments $\left(D_{3}\right)$ and

(4) progress of science and technology $\left(D_{4}\right)$.

To avoid the conflict between them, the decision makers gives the weight $\beta ={\left(0.314,0.355,0.331\right)}^{T}.$ Construct the spherical fuzzy set decision making matrices as shown in Table 1, Table 2 and Table 3:

Since $D_{1}$, $D_{3}$ are benefit type criteria and $D_{2},$ $D_{4}$ is cost type criteria, we need to have normalized the decision matrices. Normalized decision matrices are shown in Table 4, Table 5 and Table 6:

**Step 2:** Use the SFWA operator to aggregate all the individual normalized spherical fuzzy decision matrices. The aggregated spherical fuzzy decision matrix is shown in Table 7.

**Step 3**: The entropy of each attribute can be calculated by the following equation:

$$\gamma_{q}=\frac{1+\frac{1}{n}\sum_{\rho =1}^{n}\left(P_{i}\log\left(P_{i}\right)+I_{i}\log\left(I_{i}\right)+N_{i}\log\left(N_{i}\right)\right)}{\sum_{q=1}^{n}\left(1+\frac{1}{n}\sum_{\rho =1}^{n}P_{i}\log\left(P_{i}\right)+I_{i}\log\left(I_{i}\right)+N_{i}\log\left(N_{i}\right)\right)}$$

and the calculated weights are

$$W=\left\{w_{1}=0.256,w_{2}=0.248,w_{3}=0.245,w_{4}=0.251\right\}.$$

**Step 4**: Now, we apply all the proposed logarithmic aggregation operators to collective spherical fuzzy information to find the aggregated information as follows:

**Case-1**: *Using logarithmic spherical fuzzy weighted averaging aggregation operator, we obtained*


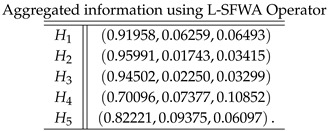


**Case-2**: *Using a logarithmic spherical fuzzy ordered weighted averaging aggregation operator, we obtained*


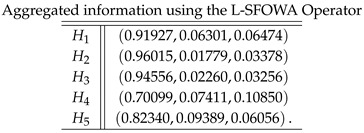


**Case-3**: *Using a logarithmic spherical fuzzy hybrid weighted averaging aggregation operator, we obtained*


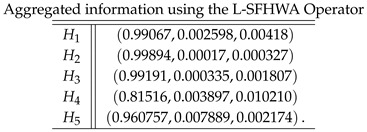


**Case-4**: *Using a logarithmic spherical fuzzy weighted geometric aggregation operator, we obtained*


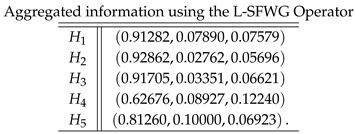


**Case-5**: *Using a logarithmic spherical fuzzy ordered weighted geometric aggregation operator, we obtained*


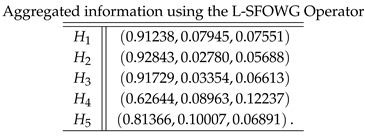


**Case-6**: *Using a logarithmic spherical fuzzy hybrid weighted geometric aggregation operator, we obtained*


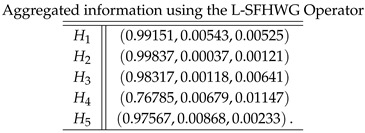


**Step 5**: We find the score value $\tilde{S}\left(\ell og_{\sigma }\varepsilon_{\rho }\right)$ and the accuracy value $\tilde{A}\left(\ell og_{\sigma }\varepsilon_{\rho }\right)$ of the cumulative overall preference value $h_{i}$ (i=1,2,3,4,5).

**Case-1**: *Score of aggregated information for the L-SFWA Operator, and we obtained*


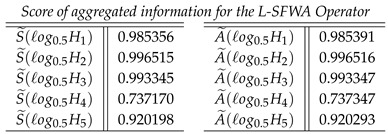


**Case-2**: *Score of aggregated information for the L-SFOWA Operator, and we obtained*


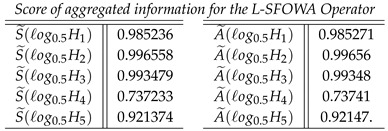


**Case-3**: *Score of aggregated information for the L-SFHWA Operator, and we obtained*


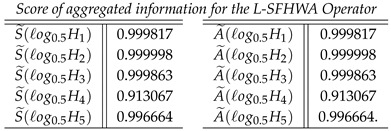


**Case-4**: *Score of aggregated information for the L-SFWG Operator, and we obtained*


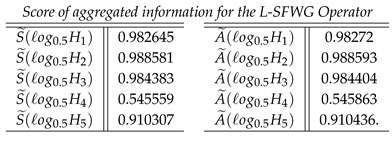


**Case-5**: *Score of aggregated information for the L-SFOWG Operator, and we obtained*


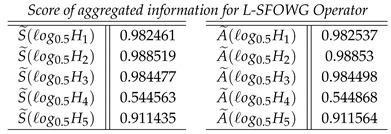


**Case-6**: *Score of aggregated inf ormation for the L-SFHWG Operator, and we obtained*


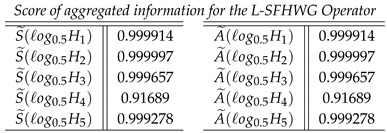


**Step 6**: We find the best (suitable) alternative that has the maximum score value from set of alternatives $h_{i}$(i=1,2,3,4,5). Overall preference value and ranking of the alternatives are summarized in Table 8.

**Figure 5:**
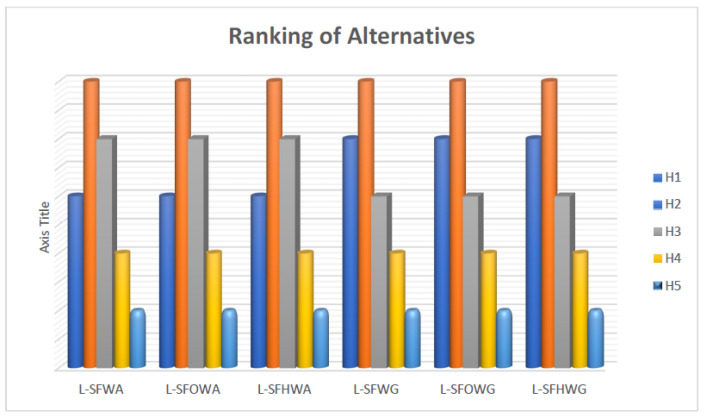
Ranking of Alternatives.

The ranking of the alternatives are shown in the Figure 5.

### 6.2. Sensitivity Analysis and Comparison Discussion

In this section, we give the comparison analysis on how our proposed logarithmic aggregation operators are effective and reliable to aggregate the spherical fuzzy information. Ashraf [40,41] proposed the spherical aggregation operators to aggregate the spherical fuzzy informatio; in this part of our study, we give a comparison between proposed and novel logarithmic spherical fuzzy aggregation operators. We take the spherical fuzzy information from [40] as follows:


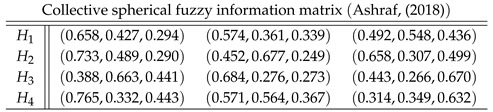


Now, we utilized a logarithmic spherical fuzzy weighted averaging operator to choose the best alternative as follows:


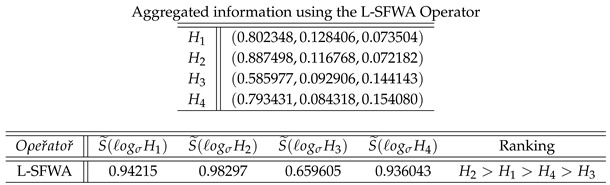


**Figure 6:**
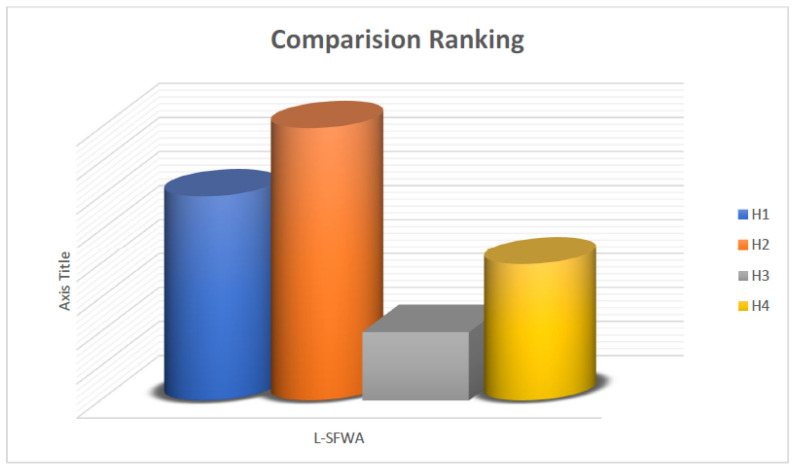
Comparison Ranking.

The ranking of the alternatives using Ashraf [40] information is shown in the Figure 6.

The bast alternative is $H_{2}.$ The obtained result utilizing a logarithmic spherical fuzzy weighted averaging operator is the same as results shown by Ashraf [40]. Hence, this study proposed the novel logarithmic aggregation operators to aggregate the spherical fuzzy information. This study gives a more reliable technique to aggregate and to deal with uncertainties in decision-making problems using spherical fuzzy sets. Utilizing proposed spherical fuzzy logarithmic aggregation operators, we find the best alternative from a set of alternatives given by the decision maker. Hence, the proposed MCGDM technique based on spherical fuzzy logarithmic aggregation operators gives another technique to find the best alternative as an application in decision support systems.

## 7. Conclusions

In this paper, we have revealed a novel logarithmic operation of SFSs with the real base number σ. Additionally, we have analyzed their properties and relationships. In view of these logarithmic operations, we built up the weighted averaging and geometric aggregation operators named L-SFWA, L-SFOWA, L-SFHWA, L-SFWG, L-SFOWG and L-SFHWG. A spherical fuzzy MCDM problem with interactive criteria, an approach based on the proposed operators, was proposed. Finally, this method was applied to MCDM problems. From the decision results displayed in numerical examples, we can find that our created approach can overcome the drawbacks of existing algebraic aggregation operators. In the succeeding work, we shall combine the proposed operator with some novel fuzzy sets, such as type-2 fuzzy sets, neutrosophic sets, and so on. In addition, we may investigate our created approach in the field of different areas, such as personnel evaluation, medical artificial intelligence, energy management and supplier selection evaluation. In addition, we can develop more decision-making approaches like GRA, TODAM, TOPSIS, VIKOR and so on to deal with uncertainties in the form of spherical fuzzy information. 

## Figures and Tables

**Table 1:** Emerging technology Eenterprises $\;F^{1}$.

	$D_{1}$	$D_{2}$	$D_{3}$	$D_{4}$
$H_{1}$	$\left(0.84,0.34,0.40\right)$	$\left(0.43,0.39,0.78\right)$	$\left(0.67,0.50,0.30\right)$	$\left(0.31,0.21,0.71\right)$
$H_{2}$	$\left(0.60,0.11,0.53\right)$	$\left(0.23,0.35,0.59\right)$	$\left(0.72,0.31,0.41\right)$	$\left(0.11,0.25,0.82\right)$
$H_{3}$	$\left(0.79,0.19,0.39\right)$	$\left(0.11,0.21,0.91\right)$	$\left(0.71,0.41,0.13\right)$	$\left(0.34,0.25,0.51\right)$
$H_{4}$	$\left(0.63,0.51,0.13\right)$	$\left(0.49,0.33,0.42\right)$	$\left(0.61,0.43,0.45\right)$	$\left(0.49,0.37,0.59\right)$
$H_{5}$	$\left(0.57,0.36,0.29\right)$	$\left(0.50,0.15,0.60\right)$	$\left(0.70,0.32,0.40\right)$	$\left(0.33,0.44,0.65\right).$

**Table 2:** Emerging technology enterprises $\;F^{2}$.

	$D_{1}$	$D_{2}$	$D_{3}$	$D_{4}$
$H_{1}$	$\left(0.61,0.15,0.53\right)$	$\left(0.16,0.35,0.62\right)$	$\left(0.61,0.35,0.47\right)$	$\left(0.55,0.17,0.74\right)$
$H_{2}$	$\left(0.66,0.11,0.51\right)$	$\left(0.43,0.23,0.77\right)$	$\left(0.93,0.08,0.09\right)$	$\left(0.02,0.06,0.99\right)$
$H_{3}$	$\left(0.88,0.09,0.07\right)$	$\left(0.05,0.06,0.89\right)$	$\left(0.56,0.17,0.44\right)$	$\left(0.43,0.13,0.61\right)$
$H_{4}$	$\left(0.59,0.32,0.34\right)$	$\left(0.24,0.48,0.51\right)$	$\left(0.68,0.53,0.39\right)$	$\left(0.34,0.21,0.61\right)$
$H_{5}$	$\left(0.71,0.31,0.24\right)$	$\left(0.35,0.41,0.69\right)$	$\left(0.73,0.44,0.21\right)$	$\left(0.22,0.49,0.74\right)$

**Table 3:** Emerging technology enterprises $\;F^{3}$.

	$D_{1}$	$D_{2}$	$D_{3}$	$D_{4}$
$H_{1}$	$\left(0.85,0.25.0.15\right)$	$\left(0.14,0.23,0.88\right)$	$\left(0.78,0.38,0.18\right)$	$\left(0.29,0.39,0.83\right)$
$H_{2}$	$\left(0.94,0.04,0.07\right)$	$\left(0.39,0.19,0.61\right)$	$\left(0.63,0.18,0.35\right)$	$\left(0.48,0.49,0.56\right)$
$H_{3}$	$\left(0.73,0.13,0.46\right)$	$\left(0.19,0.39,0.88\right)$	$\left(0.87,0.35,0.18\right)$	$\left(0.41,0.13,0.81\right)$
$H_{4}$	$\left(0.82,0.12,0.43\right)$	$\left(0.55,0.21,0.63\right)$	$\left(0.53,0.33,0.47\right)$	$\left(0.46,0.23,0.51\right)$
$H_{5}$	$\left(0.61,0.33,0.29\right)$	$\left(0.28,0.41,0.63\right)$	$\left(0.74,0.34,0.14\right)$	$\left(0.37,0.32,0.65\right)$

**Table 4:** Emerging technology enterprises $\;{\overset{ˇ}{r}}^{1}$.

	$D_{1}$	$D_{2}$	$D_{3}$	$D_{4}$
$H_{1}$	$\left(0.84,0.34,0.40\right)$	$\left(0.78,0.39,0.43\right)$	$\left(0.67,0.50,0.30\right)$	$\left(0.71,0.21,0.31\right)$
$H_{2}$	$\left(0.60,0.11,0.53\right)$	$\left(0.59,0.35,0.23\right)$	$\left(0.72,0.31,0.41\right)$	$\left(0.82,0.25,0.11\right)$
$H_{3}$	$\left(0.79,0.19,0.39\right)$	$\left(0.91,0.21,0.11\right)$	$\left(0.71,0.41,0.13\right)$	$\left(0.51,0.25,0.34\right)$
$H_{4}$	$\left(0.63,0.51,0.13\right)$	$\left(0.42,0.33,0.49\right)$	$\left(0.61,0.43,0.45\right)$	$\left(0.59,0.37,0.49\right)$
$H_{5}$	$\left(0.57,0.36,0.29\right)$	$\left(0.60,0.15,0.50\right)$	$\left(0.70,0.32,0.40\right)$	$\left(0.65,0.44,0.33\right)$

**Table 5:** Emerging technology enterprises $\;{\overset{ˇ}{r}}^{2}$.

	$D_{1}$	$D_{2}$	$D_{3}$	$D_{4}$
$H_{1}$	$\left(0.61,0.15,0.53\right)$	$\left(0.62,0.35,0.16\right)$	$\left(0.61,0.35,0.47\right)$	$\left(0.74,0.17,0.55\right)$
$H_{2}$	$\left(0.66,0.11,0.51\right)$	$\left(0.77,0.23,0.43\right)$	$\left(0.93,0.08,0.09\right)$	$\left(0.99,0.06,0.02\right)$
$H_{3}$	$\left(0.88,0.09,0.07\right)$	$\left(0.89,0.06,0.05\right)$	$\left(0.56,0.17,0.44\right)$	$\left(0.61,0.13,0.43\right)$
$H_{4}$	$\left(0.59,0.32,0.34\right)$	$\left(0.51,0.48,0.24\right)$	$\left(0.68,0.53,0.39\right)$	$\left(0.61,0.21,0.34\right)$
$H_{5}$	$\left(0.71,0.31,0.24\right)$	$\left(0.69,0.41,0.35\right)$	$\left(0.73,0.44,0.21\right)$	$\left(0.74,0.49,0.22\right)$

**Table 6:** Emerging technology enterprises $\;{\overset{ˇ}{r}}^{3}$.

	$D_{1}$	$D_{2}$	$D_{3}$	$D_{4}$
$H_{1}$	$\left(0.85,0.25.0.15\right)$	$\left(0.88,0.23,0.14\right)$	$\left(0.78,0.38,0.18\right)$	$\left(0.83,0.39,0.29\right)$
$H_{2}$	$\left(0.94,0.04,0.07\right)$	$\left(0.61,0.19,0.39\right)$	$\left(0.63,0.18,0.35\right)$	$\left(0.56,0.49,0.48\right)$
$H_{3}$	$\left(0.73,0.13,0.46\right)$	$\left(0.88,0.39,0.19\right)$	$\left(0.87,0.35,0.18\right)$	$\left(0.81,0.13,0.41\right)$
$H_{4}$	$\left(0.82,0.12,0.43\right)$	$\left(0.63,0.21,0.55\right)$	$\left(0.53,0.33,0.47\right)$	$\left(0.51,0.23,0.46\right)$
$H_{5}$	$\left(0.61,0.33,0.29\right)$	$\left(0.63,0.41,0.28\right)$	$\left(0.74,0.34,0.14\right)$	$\left(0.65,0.32,0.37\right)$

**Table 7:** Collective spherical fuzzy decision information matrix $\;\overset{ˇ}{r}$.

	$D_{1}$	$D_{2}$	$D_{3}$	$D_{4}$
$H_{1}$	$\left(0.788,0.229,0.319\right)$	$\left(0.785,0.315,0.208\right)$	$\left(0.696,0.402,0.297\right)$	$\left(0.767,0.239,0.371\right)$
$H_{2}$	$\left(0.807,0.078,0.279\right)$	$\left(0.674,0.246,0.342\right)$	$\left(0.818,0.160,0.227\right)$	$\left(0.919,0.188,0.097\right)$
$H_{3}$	$\left(0.814,0.128,0.223\right)$	$\left(0.893,0.165,0.099\right)$	$\left(0.748,0.284,0.223\right)$	$\left(0.677,0.159,0.393\right)$
$H_{4}$	$\left(0.702,0.267,0.271\right)$	$\left(0.533,0.324,0.395\right)$	$\left(0.615,0.424,0.433\right)$	$\left(0.573,0.258,0.421\right)$
$H_{5}$	$\left(0.639,0.331,0.271\right)$	$\left(0.644,0.298,0.363\right)$	$\left(0.724,0.365,0.224\right)$	$\left(0.685,0.411,0.296\right)$

**Table 8:** Overall preference value and ranking of the alternatives for σ=0.5>0.

	$\tilde{S}\left(\ell og_{\sigma }H_{1}\right)$	$\tilde{S}\left(\ell og_{\sigma }H_{2}\right)$	$\tilde{S}\left(\ell og_{\sigma }H_{3}\right)$	$\tilde{S}\left(\ell og_{\sigma }H_{4}\right)$	$\tilde{S}\left(\ell og_{\sigma }H_{5}\right)$	Ranking
*L-SFWA*	0.985356	0.996515	0.993345	0.737170	0.920198	$H_{2}>H_{3}>H_{1}>H_{5}>H_{4}$
*L-SFOWA*	0.985236	0.996558	0.993479	0.737233	0.921374	$H_{2}>H_{3}>H_{1}>H_{5}>H_{4}$
*L-SFHWA*	0.999817	0.999998	0.999863	0.913067	0.996664	$H_{2}>H_{3}>H_{1}>H_{5}>H_{4}$
*L-SFWG*	0.982645	0.988581	0.984383	0.545559	0.910307	$H_{2}>H_{3}>H_{1}>H_{5}>H_{4}$
*L-SFOWG*	0.982461	0.988519	0.984477	0.544563	0.911435	$H_{2}>H_{1}>H_{3}>H_{5}>H_{4}$
*L-SFHWG*	0.999914	0.999997	0.999657	0.91689	0.999278	$H_{2}>H_{1}>H_{3}>H_{5}>H_{4}$
